# Carbapenem-Resistant* Klebsiella pneumoniae* Infections among ICU Admission Patients in Central China: Prevalence and Prediction Model

**DOI:** 10.1155/2019/9767313

**Published:** 2019-03-27

**Authors:** Yi Li, Hui Shen, Cheng Zhu, Yuetian Yu

**Affiliations:** ^1^Department of Clinical Laboratory, Henan Provincial People's Hospital, Zhengzhou, 450003, China; ^2^Department of Laboratory Medicine, Shanghai East Hospital, Tongji University School of Medicine, Shanghai, 200123, China; ^3^Department of Emergency, Ruijin Hospital, School of Medicine, Shanghai Jiao Tong University, Shanghai, 200005, China; ^4^Department of Critical Care Medicine, Renji Hospital, School of Medicine, Shanghai Jiao Tong University, Shanghai, 200001, China

## Abstract

**Objective:**

To investigate the prevalence of infections due to carbapenem-resistant* Klebsiella pneumoniae* (CRKP) among ICU admission patients in central China and develop a reliable prediction model.

**Methods:**

Five hundred and seven consecutive ICU admission patients with* Klebsiella pneumoniae *(KP) infection were enrolled in this retrospective multicenter case-control study from January 2014 to June 2018. The prevalence and antimicrobial susceptibility pattern were analyzed. Multivariate analysis was performed by logistic regression modeling to determine the risk factors. A prediction model was developed and verified using data from six hospitals in central China.

**Results:**

Of the total 507 isolates of KP, 244 (48.1%) strains were carbapenem resistant. The majority of these isolates were from sputum (30.9%) and blood (20.9%) samples. Tigecycline had good activity against CRKP (95.5%). The most common sequence type (ST) of CRKP was ST11 (84.4%), and 98.6% of them had the* bla*KPC-2 antimicrobial resistance gene. Thirteen variables were identified as independent risk factors for CRKP infection, including KP colonization or infection in the preceding year (OR=3.32, 95% CI 2.01-4.38), CD4/CD8 ratio <1 (OR=2.98, 95% CI 2.02-4.19), and parenteral nutrition *⩾*48 h (OR=1.88, 95% CI 1.22-3.04). The model developed to predict CRKP infection was effective, with an area under the receiver-operating characteristic curve of 0.854 (95% CI 0.821-0.884,* p*<0.001).

**Conclusions:**

ST11 carrying the* bla*KPC-2 antimicrobial resistance gene was the most common type of CRKP among the ICU admission patients in central China. The model demonstrated excellent predictive performance and exhibited good discrimination.

## 1. Introduction

Infectious disease is one of the leading causes of mortality and morbidity in intensive care unit (ICU) admission patients, and multidrug-resistant (MDR) bacteria isolates are not uncommon among them [1]. Of these MDR pathogens, carbapenem-resistant* Klebsiella pneumoniae* (CRKP) poses a significant threat to public and clinical health due to its high levels of resistance to most alternative antibiotics.

Despite improvements in hospital infection control and antimicrobial scientific stewardship, CRKP is still on the rise [2]. According to reports from the Chinese drug-resistant bacteria surveillance system, a marked increase of resistance to meropenem and imipenem was seen in* Klebsiella pneumoniae*, from 2.6% to 13.4% and from 2.4% to 10.5% in the last decade, respectively [3]. Even more serious is the rate of CRKP isolation, which was 23.2% in Henan province (approximate population of 94,800,000) in 2016, with an upward trend, especially among ICU admission patients in central China [3]. One pivotal factor in managing CRKP infection is the prediction of its occurrence. Thus, a reliable prediction model with high accuracy may help to prevent or reduce the risk of CRKP infection in critical patients.

This multicenter study was performed in Henan province of central China to investigate the prevalence and risk factors of CRKP infection in ICU admission patients and to develop a reliable prediction model of CRKP infection.

## 2. Materials and Methods

### 2.1. Research Briefs

This case-control study was performed in the ICU department of 6 teaching hospitals in Henan province in central China, with a combined total of 182 ICU hospital beds. The annual volume of patients admitted in these 6 ICUs was 2,400. The 6 participating units were Henan Provincial People's Hospital, Qinyang People's Hospital, Xinzheng People's Hospital, Xuchang People's Hospital, Yongcheng People's Hospital, and the First People's Hospital of Zhumadian, all of which belong to the Henan drug-resistant bacteria surveillance association.

### 2.2. Study Population and Diseases Definition

Critically ill patients admitted to an ICU were eligible for study enrollment from January 2014 to December 2016 in Henan Provincial People's Hospital, Qinyang People's Hospital, and Xinzheng People's Hospital. Patients were included if they were (1) diagnosed with an infectious disease caused by* Klebsiella pneumoniae* and (2) between 18 and 80 years of age. Patients were excluded from the study if (1) they had more than one pathogen isolated during their ICU stay and (2) their medical history was incomplete. The validation cohort was enrolled in Xuchang People's Hospital, Yongcheng People's Hospital, and the First People's Hospital of Zhumadian from January 2017 to June 2018. The inclusion and exclusion criteria were identical to those applied to derivation cohort.

The infectious diseases included bacteremia, pneumonia, skin and soft tissue infection, urinary tract infection, and abdominal infection. The diagnostic criteria of the European Society of Clinical Microbiology and Infectious Diseases (ESCMID) were applied to diagnose these infectious diseases [4]. The presence of* Klebsiella pneumoniae* colonization was defined as any patient who had* Klebsiella pneumoniae*-positive culture results from a rectal swab screening and no clinical infection symptoms were found [4]. Nosocomial-acquired infection was defined as infectious diseases acquired after 48 h of hospitalization [5]. Patients included in the study were categorized into the CRKP group and the carbapenem-susceptible* Klebsiella pneumoniae *(CSKP) group (control group).

### 2.3. Data Collection and Clinical Assessment

Information of the patients enrolled in the study was obtained from each hospital's electronic medical records, while the antimicrobial susceptibility results were collected from the microbiology labs. The criterion for variable selection was in accordance with previous studies and specifically related to ICU patients [1–3]. The clinical characteristics of each patient were composed of three parts: (1) basic information, including age, sex, comorbidity, body mass index (BMI), history of nursing home residence, and history of* Klebsiella pneumoniae* colonization or infection in the preceding year (both the susceptible and the resistant strains were included); (2) status at ICU admission, including acute physiology and chronic health evaluation II (APACHE II) score, sequential organ failure assessment (SOFA) score, t-lymphocyte subsets, therapeutic devices, and previously performed procedures; and (3) antibiotic prescriptions within 30 days prior to* Klebsiella pneumoniae* infection.

### 2.4. Strain Identification and Antimicrobial Susceptibility Testing

Strains were identified using a bioMérieux Vitek-2 automated system (Marcy-l'Étoile, France) and confirmed by matrix-assisted laser desorption ionization time-of-flight mass spectrometry (MALDI-TOF MS, Bruker Microflex LT, Bruker Daltonik GmbH, Bremen, Germany). The presence of carbapenem resistance genes (*bla*VIM,* bla*OXA-48,* bla*KPC-2,* bla*NDM, and* bla*IMP) was detected using polymerase chain reaction (PCR). All positive PCR products were sequenced and compared with the reference sequences in the GenBank database (http://www.ncbi.nlm.nih.gov/genbank). The sequence type was identified by multilocus sequence typing (MLST).

Antimicrobial susceptibility testing was performed, and the breakpoint (susceptible, intermediate, or resistant) was interpreted according to Enterobacteriaceae M100-S27 provided by the Clinical and Laboratory Standards Institute (CLSI) standards (http://ncipd.org/control/images/NCIPD_docs/CLSI_M100-S27.pdf). Susceptibility to tigecycline was interpreted according to the 2017 European Committee on Antimicrobial Susceptibility Testing (EUCAST) breakpoints (http://www.eucast.org/fileadmin/src/media/PDFs/EUCAST_files/Breakpoint_tables/v_7.1_Breakpoint_Tables.pdf). The antibiotic susceptibility tests were conducted for piperacillin, ampicillin-sulbactam, piperacillin-tazobactam, ciprofloxacin, levofloxacin, cefuroxime, ceftazidime, cefepime, aztreonam, amikacin, gentamicin, fosfomycin, trimethoprim-sulfamethoxazole, ertapenem, meropenem, imipenem, and tigecycline. Although colistin was recommended to treat CRKP infection by EUCAST, it was not available in mainland China because of its severe side effects such as neuromuscular blockade. Thus, colistin susceptibility was not routinely tested or included in our study.

CRKP was defined as isolated* Klebsiella pneumoniae* strains that were resistant to at least one of the carbapenem agents, including ertapenem, meropenem, or imipenem [6].* Klebsiella pneumoniae* ATCC700603 was used as the quality control strain for the antibiotic susceptibility tests. To avoid duplicate counts, only the first strain was included for every patient, based on the ID number.

### 2.5. Statistical Analysis

Statistical analysis was performed using SPSS version 22.0 (IBM for Windows). The CRKP and CSKP data were compared using the chi-squared test for equal proportion or Fisher's exact test (where numbers were small), with results presented as percentages (n). Normally distributed variables were compared using Student's t-test and were expressed as the means (standard deviations), whereas nonnormally distributed data were compared using the Wilcoxon rank-sum test and were reported as medians (interquartile range). Risk factors associated with CRKP infection were identified by multivariate logistic regression and summarized with odds ratios (ORs) and 95% confidence intervals (CIs). These risk factors were incorporated into the prediction model, and the performance of the model was displayed as the area under the curve (AUC) of the receiver-operating characteristic curve (ROC). A two-sided* p*<0.05 was considered to be statistically significant. Figures were drawn using GraphPad Prism version 8.0 and Medical calculator version 15.0.

## 3. Results

### 3.1. Prevalence and Antibiotic Susceptibility of Klebsiella pneumonia

Of the total 507 isolates of* Klebsiella pneumoniae,* 244 strains (48.1%) were confirmed to be carbapenem resistant. Among these CRKP strains, more than half were isolated from respiratory specimens (62.9%), including 75 (30.9%) from sputum, 38 (15.6%) from endotracheal aspirate (ETA), and 40 (16.4%) from bronchoalveolar lavage fluid (BALF). Blood samples were another important source of CRKP, comprising 20.9% of the total amount. No significant differences were found in the source of samples between the two groups (*p*>0.05, Table S1).

The susceptibility data of* Klebsiella pneumoniae* is summarized in Table S2. Tigecycline was still the best choice of CRKP, with high susceptibility (95.5%). Other options might be amikacin (31.1%) and fosfomycin (35.2%). However, the susceptibility to these antibiotics was only slightly higher than 30%. All of the antibiotics tested in the CSKP group showed better susceptibility (*⩾*65%), except for piperacillin (55.9%) and ampicillin-sulbactam (55.9%).

### 3.2. Sequence Type and Antibiotic Resistance Gene Identification

Sequence type was identified by MLST, and the vast majority of CRKP were ST11 (n=206, 84.4%). Additionally, 12 strains of ST15, 11 strains of ST323, and 6 strains of ST1869 were also detected. Less than 5 strains, each of ST722, ST1647, ST709, and ST45, were detected. The PCR results revealed that* bla*KPC-2 was a major contributor to carbapenem resistance (96.6% in ST11, 50% in ST15, and 54.5% in ST323).* bla*NDM was mostly detected in ST15 (16.7%), ST323 (18.2%), and ST722 (50%).* bla*OXA-48 and* bla*VIM were not detected in any of the 244 CRKP isolates (Table S3).

### 3.3. Clinical Features of CRKP and CSKP Groups

In total, 507 ICU patients with infectious* Klebsiella pneumoniae *were enrolled in the study, 48.1% of whom were in the CRKP group, while the other 263 were in the CSKP group. Sex, BMI, and the number of bedridden patients did not reveal significant difference between the two groups (*p*>0.05). More patients in the CRKP group were from nursing homes (17.2% vs 6.5%), and a large portion of CRKP-infected patients currently had* Klebsiella pneumoniae* colonization or had* Klebsiella pneumoniae* infection in the preceding year (17.2% vs 6.1%) and had other resistant bacteria colonization or infection in the preceding year (15.2% vs 4.2%),* p*<0.001. The t-lymphocyte subset counts were similar between the two groups, while there was a high proportion of CD4/CD8 ratio <1 in the CRKP group (27.5% vs 7.9%,* p*<0.001).

Regarding ICU admission status and treatment procedures performed, we found that the APACHE II score was higher in the CRKP group (63.3% of patients >15 points,* p*<0.001). Patients with septic shock and those undergoing radiotherapy or chemotherapy were similar (*p*>0.05), whereas more patients in the CRKP group had undergone immunosuppressive therapy or corticosteroid therapy (*p*<0.001). Additionally, more patients in the CRKP group were found with invasive mechanical ventilation ≥48 h or with central venous catheter ≥48 h and parenteral nutrition ≥48 h (Table 1).

The relationship between CRKP/CSKP status and antibiotic (15 types) prescription in the 30 days prior to being infected by* Klebsiella pneumoniae *is shown in Table S4. Statistically significant higher exposure to aminoglycosides (19.3% vs 9.9%), carbapenems (21.7% vs 4.6%), quinolones (9.4% vs 4.2%), third-generation cephalosporins (8.6% vs 3.0%), and fourth-generation cephalosporins (11.1% vs 4.2%) was found in the CRKP group compared to the CSKP group (all* p*<0.05).

**Figure 1:**
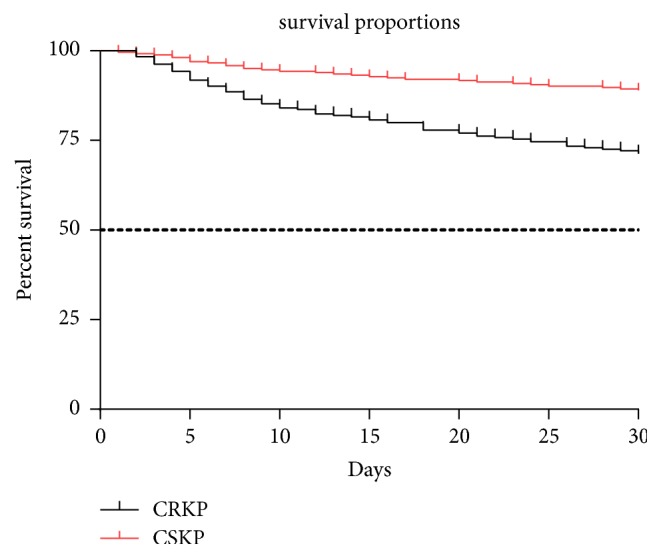
Survival proportions of the patients with CRKP or CSKP infection during 30 day ICU treatment. The dashed black line refers to 50% of survival (median survival reference line).

The 30-day mortality was significantly higher in the CRKP group—almost threefold greater than that in the CSKP group (28.9% vs 11.0%,* p*<0.001). The Kaplan-Meier survival curves revealed that most of the deaths occurred within the first 10 days of ICU admission (36 vs 14,* p*<0.05) (Figure 1).

### 3.4. Risk Factors for CRKP Infection

**Figure 2:**
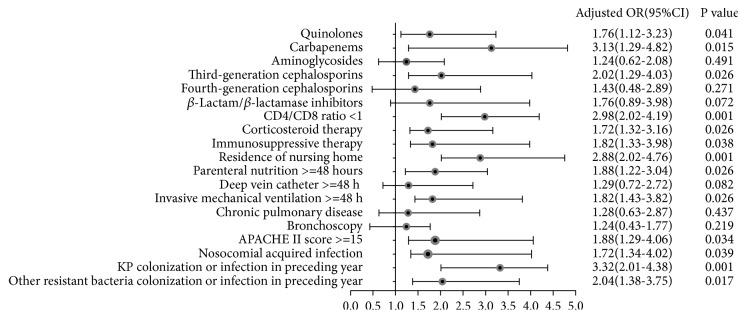
*Multivariate logistic regression analysis of risk factors for CRKP infection*.* Klebsiella pneumoniae* colonization or infection in preceding year, carbapenems exposure, and residence of nursing home were revealed as the top three risk factors as well as the other 8 risk factors. Aminoglycosides and fourth-generation cephalosporins prescription proved to be statistically nonsignificant.

Risk factors for CRKP infection were analyzed in all the enrolled patients. Based on the analysis of the baseline between the two groups, variables with* p*>0.05 were removed and eighteen variables with* p*<0.05 were incorporated into the logistic regression model. As the results of the multivariate logistic regression model revealed, thirteen variables with* p*<0.05 were retained in the final model (Figure 2).* Klebsiella pneumoniae* colonization or infection in the preceding year (OR=3.32, 95% CI 2.01-4.38), as well as other resistant bacteria colonization or infection in the preceding year (OR=2.04, 95% CI 1.38-3.75), was found to be risk factor for the development of CRKP infection (*p*<0.05). Invasive mechanical ventilation ≥48 h (OR=1.82, 95% CI 1.43-3.82) and parenteral nutrition ≥48 h (OR=1.88, 95% CI 1.22-3.04) were also confirmed as risk factors. Aminoglycosides and fourth-generation cephalosporin prescriptions proved to be statistically nonsignificant (*p*>0.05).

### 3.5. Prediction Model for CRKP Infections

In total, 507 ICU admission patients in Henan Provincial People's Hospital, Qinyang People's Hospital, and Xinzheng People's Hospital from January 2014 to December 2016 were enrolled in the derivation cohort, while 335 ICU admission patients in Xuchang People's Hospital, Yongcheng People's Hospital, and the First People's Hospital of Zhumadian from January 2017 to June 2018 were enrolled in the validation cohort.

A prediction model for CRKP infection was developed based on the thirteen risk factors that were confirmed by a multivariate logistic regression model, and the performance of the prediction model was assessed by AUC of ROC.  Table 2 presents the distribution of the cumulative risk factors between the CRKP and CSKP groups. The presence of zero risk factors was found exclusively in the CSKP group (7 patients), while no patients with ≥12 risk factors were found in this group. All patients in the CRKP group had greater than 2 risk factors.

**Figure 3:**
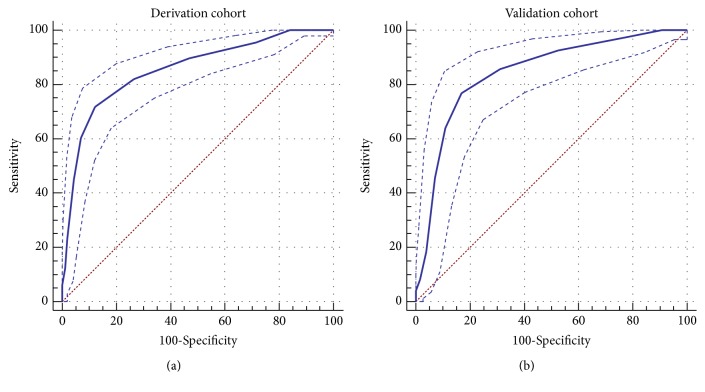
Receiver-operating characteristic curves for the predictive model. (a) Derivation set. (b) Validation set.

The ROC AUC was 0.854 (95% CI 0.821-0.884,* p*<0.001) in the derivation cohort, while in the validation cohort it was 0.844 (95% CI 0.800-0.881,* p*<0.001), indicating that the model had excellent predictive power (Figure 3).  Table 3 displays the predictive efficacy derived from the model in the derivation and validation cohorts. Diagnostic performance parameters are shown for different cutoffs. The prediction model performed best with a cutoff of ≥6 risk factors, with 82% sensitivity and 74% specificity (the entire accuracy was 78%).

## 4. Discussion

CRKP infection was first reported in northeast Scotland in 1997 [7]. Since then, it has been detected worldwide and constitutes a significant growing public health threat. The emergence of CRKP was primarily a consequence of the widespread acquisition of carbapenemase genes; unrestricted carbapenems consumption promoted the rising trend as well [8]. The prevalence of CRKP varies between countries and regions and between medical institutions [9, 10]. Although some studies have identified a series of CRKP infection risk factors in children, newborns, and pregnant women [11, 12], few studies have focused on adult ICU patients in high CRKP infection incidence areas such as Henan province in central China. During our five-year study, we found that the average CRKP isolation rate was 48.1% among ICU* Klebsiella pneumoniae *infection patients in central China, which is far beyond the average ratio in the Chinese bacterial surveillance system (13.4%) [3]. The growing trend has aroused wide public concern, and more effective counteractive measures, such as antimicrobial scientific stewardship and improved hospital infection control procedures, have been taken [13].

Combination therapy is often required in the management of CRKP infections. However, the optimal treatment is still unknown, and the selection of drugs is constrained in the ongoing antibiotic resistance crisis. Although there has been a rapid development of new antibiotics, most are not available in China, such as ceftazidime-avibactam. The susceptibility of CRKP to antibiotics was lower than expected among the ICU patients in Henan province. In our study, it appeared that only tigecycline could be a better choice (susceptibility of 95.5%); while amikacin might be an alternative, its susceptibility was only slightly higher than 30%. In recent years, fosfomycin (susceptibility of 35.2%) has been recommended as a supplement in treating CRKP infection, although the CLSI standards propose it only for the treatment of urinary tract infections. In addition, the incidence of fosfomycin resistance has markedly varied, from 0% to 97.2% due to the dissemination of the* fosA3* gene [14].

Our study demonstrated that ST11 was still the dominant clone of CRKP, and* bla*KPC-2 was the most common carbapenemase which conforms to the global epidemiology of* Klebsiella pneumoniae* carbapenemases [15]. However, unlike other countries in Asia (such as India),* bla*OXA-48 and* bla*VIM were not detected in any of the 244 CRKP isolates.

As the results revealed, the APACHE II score in more than half of the patients in the CRKP group (63.9%) was ≥15. Thus, more life support systems were needed to cure these patients in critical conditions. The mucous membranes of the skin and trachea were damaged by invasive procedures and catheter implantation, which increased the chance of contact with the CRKP strains of colonized patients or contaminated objects [13]. We found that invasive mechanical ventilation ≥48 h (OR=1.82) and parenteral nutrition ≥48 h (OR=1.88) were risk factors for CRKP infection. Therefore, unnecessary interventional apparatus in the ICU should be removed as early as possible to prevent nosocomial-acquired infection, and enteral feeding ought to be established as soon as possible. Different from other studies of extended-spectrum beta lactamase-producing Enterobacteriaceae [16], urethral catheter placement ≥48 h did not prove to be a risk factor, because the majority of the samples in our study were from respiratory specimens (62.9%).

It is widely known that infection control in the ICU is very difficult due to the suppressed immunity and critical status of the admitted patients. Accordingly, ninety-two (37.7%) patients in the CRKP group were confirmed to have a nosocomial-acquired infection. Our study indicated that there were more patients who had immunosuppressive therapy (11.1% vs 3.4%), corticosteroid therapy (14.3% vs 4.2%), or a CD4/CD8 ratio <1 (27.5% vs 7.9%) in the CRKP group than in the CSKP group (all* p*<0.05). Thus, immunity enhancement measures such as intravenous immunoglobulin or thymosin application in patients who are susceptible to infectious diseases might be an alternative option. This will be verified in our further studies.

ICU patients with infectious diseases may be in a critical condition and typically have a high mortality. Fifty-two (21.3%) patients in the CRKP group had intercurrent septic shock and required vasopressin therapy, while in the other group the rate of septic shock was also high at 17.9%. Typically, antibiotic coverage should be adequate and appropriate for any possible pathogen. However, the indiscriminate consumption of antibiotics has accelerated the incidence of antibiotic resistance in recent years [17].

The previous prescription of antibiotics was included as a CRKP risk factor in our prediction model. Our study indicated that the use of third-generation cephalosporins, quinolones, and carbapenems within 30 days before the patients were infected by* Klebsiella pneumoniae* increased the risk of CRKP infection by 2.02, 1.76, and 2.67 times, respectively. According to a clinical epidemiology meta-analysis of carbapenem-resistant Enterobacteriaceae [18], the previous use of carbapenems and quinolones increased the risk of CRKP infection; however, there was a large variation in the odds ratio. Studies enrolled in the meta-analysis showed that the previous prescription of carbapenems led to a 7-fold increase in the risk of CRKP infection, while that of quinolones was about 3-fold. The diverse odds ratios of the previous antibiotic treatments were due to the various study groups, different sample sizes, or variations in control group selection. Regarding cephalosporins, one retrospective study demonstrated that the odds ratio was 3.84 [19]. In our study, we divided the cephalosporins into four subgroups and found that only the third-generation cephalosporin was a risk factor. Further studies are required to identify the mechanism responsible for this result.

The history of* Klebsiella pneumoniae* colonization or infection in the preceding year was found to be the principal risk factor for CRKP infection, with a risk of approximately 3.3 times higher than those without a history of colonization. Meanwhile, MDR bacteria colonization or infection in the preceding year proved to be another significant risk factor (OR=2.04). Previous research has demonstrated the relationship between MDR bacteria and CRKP colonization risk [20]. This increase in subjects from which MDR pathogens were isolated or colonized might be related to overexposure to a variety of antibiotics or healthcare environments, which was also reported to be a risk factor in a previous study [21]. In this study, the isolation of MDR pathogens was found to be a risk factor for CRKP infection, even after controlling for antibiotic use and disease severity (APACHE II and SOFA), indicating that cross infection to those with other infections easily occurs.

We found some risk factors that were closely related to CRKP that had also been reported for the general population, such as nosocomial-acquired infection and a history of nursing home residence [22]. Other factors specifically associated with ICU admission patients such as therapeutic devices and procedures performed were thoroughly analyzed as well. Our predictive model included thirteen predictors of CRKP infection. If we intend to screen ICU patients to determine the possibility of infection of CRKP, a cutoff point with high sensitivity and low specificity should be adopted. The cutoff value was based on an assessment of accuracy, positive predictive value (PPV), negative predictive value (NPV), sensitivity, and specificity. In our prediction model the best cutoff value for predicting CRKP infection was ≥6 points, with a ROC AUC of 0.854 in the derivation cohort and 0.844 in the validation cohort. The model revealed excellent predictive performance and exhibited good discrimination.

We established a reliable prediction model using data from Henan province in central China and verified this model. This was the first study to identify specific risk factors for CRKP infection in ICU admission patients. However, there are some limitations. The number of patients enrolled was relatively low in our retrospective study, which limited the establishment of subgroups such as the daily dose corticosteroid therapy and the different drugs prescribed in immunosuppressive therapy. The validation cohort selected in our study was from Henan province; therefore, we could not verify whether the prediction model would be reliable in other parts of China. Thus, multicenter prospective studies with larger populations from different areas are needed to validate our findings.

In conclusion, the rate of CRKP isolation remains on the rise and has become a major threat to public health, especially to those in critical conditions. An accurate and convenient prediction model for recognizing the risk of CRKP may improve empiric antibiotic prescription and decrease the rate of treatment failure and adverse effects. Therefore, this model should be applied in screening ICU patients with infectious diseases, helping to identify the high-risk patients and provide precise antibiotics treatment.

## Figures and Tables

**Table 1:** Demographics and clinical characteristics of CRKP and CSKP groups.

Characteristics	CRKP	CSKP	*P* value
(n, % / mean±SD)	n=244	n=263	
Age, yrs	50.6±17.4	48.2±19.3	0.149
Gender (male)	132 (54.1)	135 (51.3)	0.533
BMI	23.1±4.2	24.29±5.9	0.372
Comorbidity			
Diabetes mellitus	42 (17.2)	40 (15.2)	0.54
Chronic renal failure	30 (12.3)	41 (15.6)	0.286
Chronic pulmonary disease	84 (34.4)	65 (24.7)	0.016
Hematologic disease	12 (4.92)	14 (5.32)	0.836
Nervous system disease	26 (10.7)	35 (13.3)	0.359
Charlson comorbidity index score *⩾*3	68 (27.9)	53 (22.1)	0.131
APACHE II score *⩾*15	156 (63.9)	83 (31.6)	<0.001
SOFA score	4.1±1.5	3.7±1.7	0.103
Therapeutic devices and procedures performed			
Surgery	74 (30.3)	66 (25.1)	0.188
Blood transfusion	21 (8.6)	12 (4.6)	0.065
Endoscopy	7 (2.87)	11 (4.18)	0.429
Bronchoscopy	72 (29.1)	51 (19.4)	0.007
Hemodialysis	23 (9.4)	31 (11.8)	0.389
Invasive mechanical ventilation *⩾* 48 h	86 (35.2)	50 (19.1)	<0.001
Central venous catheter *⩾* 48 h	186 (76.2)	175 (66.5)	0.016
Urethral catheter*⩾* 48 h	117 (47.9)	136 (51.7)	0.398
Parenteral nutrition *⩾* 48 h	94 (38.5)	37 (14.1)	<0.001
Hospitalization *⩾* 48 h in preceding 90 days	32 (13.1)	41 (15.6)	0.428
KP colonization or infection in the preceding year	42 (17.2)	16 (6.1)	<0.001
Other resistant bacteria colonization or infection in the preceding year	37 (15.2)	11 (4.2)	<0.001
Bedridden	21 (8.6)	19 (7.2)	0.564
Residence of nursing home	42 (17.2)	17 (6.5)	<0.001
Nosocomial acquired infection	92 (37.7)	41 (15.6)	<0.001
Immunosuppressive therapy	27 (11.1)	9 (3.4)	<0.001
Corticosteroid therapy	35 (14.3)	11 (4.2)	<0.001
Radiotherapy	4 (1.5)	4 (1.5)	0.803
Chemotherapy	3 (1.2)	4 (1.5)	0.921
Septic shock	52 (21.3)	47 (17.9)	0.329
CD4/CD8 ratio <1	67 (27.5)	21 (7.9)	<0.001
Natural killer cell (cells/uL)	191.3±57.4	200.7±54.6	0.059
B lymphocyte (cells/uL)	75.6±24.6	78.7±30.2	0.208
T lymphocyte (cells/uL)	1137.5±202.5	1108.8±185.2	0.096
Th lymphocyte (cells/uL)	338.9±80.3	348.4±82.9	0.191
Ts lymphocyte (cells/uL)	304.8±78.4	298.5±69.3	0.337
30-day mortality	70 (28.9)	29 (11.0)	<0.001

CRKP: carbapenem-resistant *Klebsiella pneumoniae*; CSKP: carbapenem susceptible *Klebsiella pneumoniae*; BMI: body mass index; APACHE II: acute physiology and chronic health evaluation II; SOFA: sequential organ failure assessment.

**Table 2:** Distribution of cumulative risk factors for *Klebsiella pneumoniae* infected patients.

Number of risk factors	Number of patients, n (%)		
	CRKP	CSKP	Total
Derivation cohort			
0	0 (0)	7 (100)	7
1	0 (0)	21 (100)	21
2	0 (0)	20 (100)	20
3	11 (25.6)	32 (74.4)	43
4	14 (18.2)	63 (81.8)	77
5	19 (26.8)	52 (73.2)	71
6	25 (40.3)	37 (59.7)	62
7	28 (68.3)	13 (31.7)	41
8	37 (84.1)	7 (15.9)	44
9	54 (90)	6 (10)	60
10	26 (92.9)	2 (7.1)	28
11	15 (83.3)	3 (16.7)	18
12	9 (100)	0 (0)	9
13	6 (100)	0 (0)	6
Total	244 (48.1)	263 (51.9)	507
Validation cohort			
0	0 (0)	5 (100)	5
1	0 (0)	7 (100)	7
2	0 (0)	10 (100)	10
3	4 (13.8)	25 (86.2)	29
4	7 (13.5)	45 (86.5)	52
5	10 (20.4)	39 (79.6)	49
6	13 (33.3)	26 (66.7)	39
7	19 (63.3)	11 (36.7)	30
8	27 (79.4)	7 (20.6)	34
9	40 (86.9)	6 (13.1)	46
10	15 (78.9)	4 (21.1)	19
11	6 (66.6)	3 (33.4)	9
12	4 (100)	0 (0)	4
13	2 (100)	0 (0)	2
Total	147 (43.9)	188 (56.1)	335

CRKP: carbapenem-resistant *Klebsiella pneumoniae*; CSKP: carbapenem susceptible *Klebsiella pneumoniae*.

**Table 3:** Performance of the models for predicting CRKP infection at different cutoff values.

No. of risk factors	TP	FP	TN	FN	Se (%)	Sp (%)	PPV (%)	NPV (%)	Acc (%)
Derivation cohort									
*⩾*1	244	256	7	0	100	3	49	100	50
*⩾*2	244	235	28	0	100	11	51	100	54
*⩾*3	244	215	48	0	100	18	53	100	58
*⩾*4	233	183	80	11	96	30	56	88	62
*⩾*5	219	120	143	25	90	54	65	85	71
*⩾*6	200	68	195	44	82	74	75	82	78
*⩾*7	175	31	232	69	72	88	85	77	80
*⩾*8	147	18	245	97	60	93	89	72	77
*⩾*9	110	11	252	134	45	96	91	65	71
*⩾*10	56	5	258	188	23	98	92	58	62
*⩾*11	30	3	260	214	12	99	91	55	57
*⩾*12	15	0	263	229	6	100	100	53	55
*⩾*13	6	0	263	238	3	100	100	52	53
Validation cohort									
*⩾*1	147	183	5	0	100	3	45	100	45
*⩾*2	147	176	12	0	100	6	46	100	47
*⩾*3	147	166	22	0	100	12	47	100	50
*⩾*4	143	141	47	4	97	25	50	92	57
*⩾*5	136	96	92	11	93	49	59	89	68
*⩾*6	126	57	131	21	86	70	69	86	77
*⩾*7	113	31	157	34	77	84	78	82	81
*⩾*8	94	20	168	53	64	89	82	76	78
*⩾*9	67	13	175	80	46	93	84	69	72
*⩾*10	27	7	181	120	18	96	79	60	62
*⩾*11	12	3	185	135	8	98	80	58	59
*⩾*12	6	0	188	141	4	100	100	57	58
*⩾*13	2	0	188	145	2	100	100	56	57

TP: number of true positives; FP: number of false positives; FN: number of false negatives; TN: number of true negatives; Se: sensitivity; Sp: specificity; PPV: positive predictive value; NPV: negative predictive value; Acc: rate of accuracy of the risk score model.

## Data Availability

The data used to support the findings of this study including the distribution of CRKP detection, antimicrobial susceptibility testing, and the sequence type were included within the supplementary information file of this research article.

## Abbreviations

AUC: Area under curve
APACHE II: Acute physiology and chronic health evaluation II
BMI: Body mass index
BALF: Bronchoalveolar lavage fluid
CIs: Confidence intervals
CLSI: Clinical and Laboratory Standards Institute
CRKP: Carbapenem-resistant* Klebsiella pneumoniae*
CSKP: Carbapenem-susceptible* Klebsiella pneumoniae*
ETA: Endotracheal aspirate
ESCMID: European Society of Clinical Microbiology and Infectious Diseases
EUCAST: European Committee on Antimicrobial Susceptibility Testing
ICU: Intensive care unit
KP: * Klebsiella pneumoniae*
MALDI-TOF MS: Matrix-assisted laser desorption ionization time-of-flight mass spectrometry
MDR: Multidrug resistance
MLST: Multilocus sequence typing
OR: Odds ratio
PCR: Polymerase chain reaction
ROC: Receiver-operating characteristic
SOFA: Sequential organ failure assessment.

## Supplementary Materials

Supplementary Materials The supplementary materials about this research article included four parts as follows: (1) Sample distribution of CRKP and CSKP; (2) antimicrobial susceptibility patterns of CRKP and CSKP strains to various antimicrobials; (3) prevalence of antimicrobial resistance gene in all the 244 carbapenem resistant* Klebsiella pneumoniae *strains in terms of ST types; (4) antibiotics prescription within 30 days before the patients were infected by* Klebsiella pneumoniae*; and (5) the data of sequence type and antimicrobial susceptibility testing.
