# Supplementary material for: Carbapenem-Resistant Klebsiella pneumoniae Infections among ICU Admission Patients in Central China: Prevalence and Prediction Model
Source: Biomed Res Int. 2019 Mar 27;2019:9767313. doi: 10.1155/2019/9767313 (PMC6457282; doi:10.1155/2019/9767313)
Supplement: Supplementary Materials — The supplementary materials about this research article included four parts as follows: (1) Sample distribution of CRKP and CSKP; (2) antimicrobial susceptibility patterns of CRKP and CSKP strains to various antimicrobials; (3) prevalence of antimicrobial resistance gene in all the 244 carbapenem resistant Klebsiella pneumoniae strains in terms of ST types; (4) antibiotics prescription within 30 days before the patients were infected by Klebsiella pneumoniae; and (5) the data of sequence type and antimicrobial susceptibility testing. [file 9767313.f1.pdf]

Supplementary Materials

The supplementary materials about this research article included four parts as follows: 1) Sample distribution of CRKP and CSKP; 2) Antimicrobial susceptibility patterns of CRKP and CSKP strains to various antimicrobials; 3) Prevalence of antimicrobial resistance gene in all the 244 carbapenem resistant *Klebsiella pneumoniae* strains in terms of ST types; 4) Antibiotics prescription within 30 days before the patients infected by *Klebsiella pneumoniae* and 5) The data of sequence type and antimicrobial susceptibility testing.

Table and figure legend

Table S1 Sample distribution of CRKP and CSKP, n (%)

Specimen type	Total<br>n=507	CRKP<br>n=244	CSKP<br>n=263	*P* value
Sputum	151 (29.8)	75 (30.9)	76 (28.8)	0.651
ETA	77 (15.2)	38 (15.6)	39 (14.9)	0.815
BALF	83 (16.4)	40 (16.4)	43 (16.5)	0.989
Urine	18 (3.6)	10 (4.3)	8 (2.9)	0.521
Blood	108 (21.3)	52 (20.9)	56 (21.5)	0.996
Pus	20 (3.9)	10 (4.1)	10 (3.8)	0.864
Wound secretion	50 (9.8)	19 (7.8)	31 (11.6)	0.131

ETA, Endotracheal aspirate; BALF, Bronchoalveolar Lavage Fluid; CRKP, Carbapenem-resistant *Klebsiella pneumoniae*; CSKP, Carbapenem susceptible *Klebsiella pneumoniae;*

Table S2 Antimicrobial susceptibility patterns of CRKP and CSKP strains to various antimicrobials, n (%)

Antibiotics	Total<br>n=507	CRKP<br>n=244	CSKP<br>n=263
Piperacillin	147 (28.9)	0 (0)	147 (55.9)
Ampicillin-sulbactam	147 (28.9)	0 (0)	147 (55.9)
Piperacillin-tazobactam	228 (44.9)	0 (0)	228 (86.7)
cCiprofloxacin	206 (40.6)	7 (2.9)	199 (75.7)
Levofloxacin	215 (42.4)	9 (3.7)	206 (78.3)
Cefuroxime	173 (34.1)	1 (0.4)	172 (65.4)
Ceftazidime	207 (40.8)	3 (1.2)	204 (77.6)
Cefepime	195 (38.5)	4 (1.6)	191 (72.6)
Aztreonam	187 (36.9)	0 (0)	187 (71.1)
Amikacin	326 (64.3)	76 (31.1)	250 (95.1)
Gentamicin	218 (42.9)	28 (11.5)	190 (72.2)
Fosfomycin	281 (55.4)	86 (35.2)	195 (74.1)
Tigecycline	488 (96.3)	233 (95.5)	255 (96.9)
Trimethoprim-sulfamethoxazole	200 (39.4)	0 (0)	200 (76.0)
Ertapenem	263 (51.9)	0 (0)	263 (100)
Meropenem	267 (52.7)	4 (1.6)	263 (100)

**Imipenem**	**263 (51.9)**	**0 (0)**	**263 (100)**

CRKP, Carbapenem-resistant Klebsiella pneumoniae; CSKP, Carbapenem susceptible Klebsiella pneumoniae;

Table S3 Prevalence of antimicrobial resistance gene in all the 244 carbapenem resistant *Klebsiella pneumoniae* strains in terms of ST types, n(%)

STs	Antibiotic resistance gene				
	***bla*KPC-2**	***bla*NDM**	***bla*IMP**	***bla*OXA**	***bla*VIM**
ST11 (n=206)	199 (96.6)	0	0	0	0
ST15 (n=12)	6 (50)	2 (16.7)	1 (8.3)	0	0
ST323 (n=11)	6 (54.5)	2 (18.2)	0	0	0
ST1869 (n=6)	2 (33.3)	0	1 (16.7)	0	0
ST722 (n=4)	0	2 (50)	1 (25)	0	0
ST1647 (n=2)	0	0	1 (50)	0	0
ST709 (n=2)	1 (50)	0	0	0	0
ST45 (n=1)	0	0	0	0	0

ST, sequence type

Table S4 Antibiotics prescription within 30 days before the patients infected by *Klebsiella pneumoniae*

Prior antibiotics treatment (n, %)	CRKP n=244	CSKP n=263	*P* value
Aminoglycosides	47 (19.3)	26 (9.9)	0.004
Carbapenems	53 (21.7)	12 (4.6)	<0.001
Co-trimoxazoles	7 (2.9)	5 (1.9)	0.672
Penicillin groups	12 (4.9)	9 (3.4)	0.398
Quinolones	23 (9.4)	11 (4.2)	0.018
Macrolides	3 (1.2)	5 (1.9)	0.803
Tetracyclines	3 (1.2)	7 (2.7)	0.401
Nitroimidazoles	5 (2.0)	5 (1.9)	0.842
Fosfomycins	7 (2.9)	9 (3.4)	0.722
Glycopeptides	11 (4.5)	8 (3.0)	0.385
First-generation cephalosporins	17 (6.9)	9 (3.4)	0.07
Second-generation cephalosporins	10 (4.1)	12 (4.6)	0.798
Third-generation cephalosporins	21 (8.6)	8 (3.0)	0.012
Fourth-generation cephalosporins	27 (11.1)	11 (4.2)	0.003
β-Lactam/β-lactamase inhibitors	43 (17.6)	47 (17.9)	0.942

CRKP, Carbapenem-resistant *Klebsiella pneumoniae*; CSKP, Carbapenem susceptible *Klebsiella pneumoniae*;

Table 1							
Number	Patients ID	Gender	Department	Sample ID	Sample distribution	Bacteria	ST type
1	1211008	m	icu	1712211089	sputum	kpn	ST11
2	1255707	m	icu	170307010	sputum	kpn	ST11
3	1387145	f	icu	170701698	sputum	kpn	ST11
4	1435940	m	icu	170423020	sputum	kpn	ST11
5	1457348	f	icu	170507016	sputum	kpn	ST11
6	1467859	m	icu	1711214038	sputum	kpn	ST1869
7	1603858	f	icu	170512076	sputum	kpn	ST11
8	1628916	m	icu	1709026113	sputum	kpn	ST11
9	1631286	f	icu	1711234030	sputum	kpn	ST15
10	1643835	f	icu	170408661	sputum	kpn	ST11
11	1654306	m	icu	1709146103	sputum	kpn	ST11
12	1655141	f	icu	170226009	sputum	kpn	ST11
13	1673544	f	icu	170104515	sputum	kpn	ST11
14	1674016	m	icu	170103010	sputum	kpn	ST11
15	1676693	f	icu	170517321	sputum	kpn	ST11
16	1676967	f	icu	170316684	sputum	kpn	ST11
17	1677363	f	icu	170102017	sputum	kpn	ST323
18	1677521	f	icu	170112010	sputum	kpn	ST11
19	1678002	m	icu	170122053	sputum	kpn	ST11
20	1678087	f	icu	170905G011	sputum	kpn	ST15
21	1678329	f	icu	170101705	sputum	kpn	ST11
22	1679510	f	icu	170108647	sputum	kpn	ST11
23	1681169	m	icu	170201014	sputum	kpn	ST722
24	1681287	f	icu	170112076	sputum	kpn	ST11
25	1681407	m	icu	170224699	sputum	kpn	ST11
26	1681610	f	icu	170111060	sputum	kpn	ST1869
27	1683217	m	icu	170117042	sputum	kpn	ST11
28	1683428	f	icu	170118514	sputum	kpn	ST11
29	1683853	f	icu	170112030	sputum	kpn	ST11
30	1683893	m	icu	1711051011	sputum	kpn	ST11
31	1684044	f	icu	170225056	sputum	kpn	ST11
32	1684190	m	icu	170418324	sputum	kpn	ST11
33	1684510	f	icu	170125061	sputum	kpn	ST11
34	1684821	m	icu	170128039	sputum	kpn	ST15
35	1685440	f	icu	170125514	sputum	kpn	ST11
36	1687366	f	icu	170201055	sputum	kpn	ST11
37	1688971	f	icu	170305308	sputum	kpn	ST11
38	1689120	m	icu	170203046	sputum	kpn	ST11
39	1689894	f	icu	170330084	sputum	kpn	ST11
40	1690370	f	icu	170202637	sputum	kpn	ST709
41	1691389	m	icu	170204011	sputum	kpn	ST11
42	1692011	f	icu	170208039	sputum	kpn	ST11
43	1695674	m	icu	170210067	sputum	kpn	ST11
44	1696129	f	icu	170212012	sputum	kpn	ST11
45	1696670	m	icu	170405022	sputum	kpn	ST11
46	1698113	f	icu	170325017	sputum	kpn	ST11
47	1700328	m	icu	170319512	sputum	kpn	ST11
48	1700934	f	icu	170222047	sputum	kpn	ST11
49	1702475	m	icu	170306014	sputum	kpn	ST15
50	1703413	f	icu	170304029	sputum	kpn	ST11
51	1704192	m	icu	170620637	sputum	kpn	ST11
52	1706017	m	icu	170318057	sputum	kpn	ST11

53	1707501	f	icu	170308002	sputum	kpn	ST11
54	1707627	f	icu	170306704	sputum	kpn	ST11
55	1710844	m	icu	170423668	sputum	kpn	ST11
56	1711345	f	icu	170327045	sputum	kpn	ST11
57	1711559	m	icu	1711186131	sputum	kpn	ST11
58	1711905	m	icu	170313026	sputum	kpn	ST11
59	1712856	f	icu	170318058	sputum	kpn	ST15
60	1713261	m	icu	170314669	sputum	kpn	ST11
61	1713952	m	icu	170331075	sputum	kpn	ST11
62	1714960	f	icu	170425007	sputum	kpn	ST11
63	1715473	m	icu	170402520	sputum	kpn	ST11
64	1716196	f	icu	170331520	sputum	kpn	ST11
65	1717117	m	icu	170327009	sputum	kpn	ST11
66	1717330	f	icu	170328516	sputum	kpn	ST11
67	1718209	m	icu	170325303	sputum	kpn	ST11
68	1718292	f	icu	170401207	sputum	kpn	ST15
69	1718861	f	icu	170329537	sputum	kpn	ST11
70	1719002	f	icu	170409512	sputum	kpn	ST11
71	1719585	f	icu	170401011	sputum	kpn	ST11
72	1720545	m	icu	170411001	sputum	kpn	ST11
73	1722263	m	icu	170409012	sputum	kpn	ST15
74	1722520	m	icu	1711271077	sputum	kpn	ST11
75	1722588	f	icu	170513605	sputum	kpn	ST11
76	1722653	m	icu	170420512	ETA	kpn	ST11
77	1722820	f	icu	170402714	ETA	kpn	ST11
78	1723040	f	icu	170601046	ETA	kpn	ST11
79	1724993	m	icu	170408030	ETA	kpn	ST11
80	1725517	m	icu	170419004	ETA	kpn	ST11
81	1725783	f	icu	1711261073	ETA	kpn	ST11
82	1727646	m	icu	170417522	ETA	kpn	ST15
83	1728409	m	icu	1709161073	ETA	kpn	ST11
84	1728979	f	icu	170417049	ETA	kpn	ST11
85	1729216	f	icu	170423018	ETA	kpn	ST11
86	1729494	m	icu	170416679	ETA	kpn	ST722
87	1732456	f	icu	170427005	ETA	kpn	ST11
88	1732972	m	icu	170428036	ETA	kpn	ST11
89	1733175	f	icu	170603518	ETA	kpn	ST11
90	1733558	m	icu	170501015	ETA	kpn	ST11
91	1735030	f	icu	170620007	ETA	kpn	ST11
92	1736316	m	icu	170517054	ETA	kpn	ST11
93	1736478	f	icu	170520665	ETA	kpn	ST323
94	1736545	f	icu	170503060	ETA	kpn	ST11
95	1736849	m	icu	170518070	ETA	kpn	ST11
96	1737908	f	icu	170525054	ETA	kpn	ST709
97	1738572	m	icu	170520020	ETA	kpn	ST11
98	1738637	f	icu	170511057	ETA	kpn	ST11
99	1739659	f	icu	170506514	ETA	kpn	ST11
100	1739709	m	icu	170511052	ETA	kpn	ST11
101	1740093	f	icu	170525062	ETA	kpn	ST11
102	1740266	m	icu	170511067	ETA	kpn	ST323
103	1741070	f	icu	170514017	ETA	kpn	ST11
104	1746779	m	icu	170529630	ETA	kpn	ST11
105	1748811	f	icu	170607072	ETA	kpn	ST15
106	1749608	m	icu	170525708	ETA	kpn	ST11

107	1749979	f	icu	170528001	ETA	kpn	ST11
108	1750181	f	icu	170603055	ETA	kpn	ST11
109	1750600	f	icu	170601010	ETA	kpn	ST11
110	1750762	f	icu	170619037	ETA	kpn	ST11
111	1751643	m	icu	170605021	ETA	kpn	ST11
112	1752526	m	icu	170615061	ETA	kpn	ST11
113	1753504	m	icu	170706022	ETA	kpn	ST323
114	1753816	m	icu	170624060	ETA	kpn	ST11
115	1755284	f	icu	170626038	BALF	kpn	ST11
116	1755340	f	icu	170703060	BALF	kpn	ST11
117	1756051	m	icu	170610062	BALF	kpn	ST11
118	1756142	f	icu	170611623	BALF	kpn	ST1869
119	1757201	m	icu	170622070	BALF	kpn	ST11
120	1757736	m	icu	170614528	BALF	kpn	ST11
121	1758330	f	icu	170615033	BALF	kpn	ST11
122	1758395	m	icu	170625620	BALF	kpn	ST11
123	1758448	m	icu	170707206	BALF	kpn	ST722
124	1759555	m	icu	170719677	BALF	kpn	ST11
125	1759817	m	icu	170705612	BALF	kpn	ST11
126	1760643	f	icu	170620731	BALF	kpn	ST11
127	1761292	f	icu	170701512	BALF	kpn	ST11
128	1761352	m	icu	170705058	BALF	kpn	ST11
129	1762617	m	icu	170701062	BALF	kpn	ST11
130	1762843	m	icu	170701061	BALF	kpn	ST11
131	1763053	m	icu	1708081001	BALF	kpn	ST323
132	1763080	m	icu	170701042	BALF	kpn	ST11
133	1765040	m	icu	170715061	BALF	kpn	ST1647
134	1765508	m	icu	170701053	BALF	kpn	ST11
135	1765923	m	icu	170706076	BALF	kpn	ST11
136	1766094	m	icu	170719028	BALF	kpn	ST11
137	1766123	m	icu	170704068	BALF	kpn	ST11
138	1766176	m	icu	170707039	BALF	kpn	ST323
139	1766198	f	icu	170801050	BALF	kpn	ST11
140	1766489	m	icu	170706077	BALF	kpn	ST11
141	1767244	m	icu	170705062	BALF	kpn	ST11
142	1767348	f	icu	170722031	BALF	kpn	ST11
143	1768100	f	icu	170705001	BALF	kpn	ST11
144	1769562	m	icu	170711078	BALF	kpn	ST45
145	1769847	f	icu	170709027	BALF	kpn	ST11
146	1770111	f	icu	170723203	BALF	kpn	ST11
147	1770177	m	icu	170710740	BALF	kpn	ST11
148	1770730	f	icu	170712065	BALF	kpn	ST11
149	1771549	f	icu	170731043	BALF	kpn	ST11
150	1772176	m	icu	1708104017	BALF	kpn	ST323
151	1772963	f	icu	170724501	BALF	kpn	ST11
152	1773213	m	icu	170714730	BALF	kpn	ST11
153	1773735	f	icu	170719055	BALF	kpn	ST11
154	1774722	m	icu	170725025	BALF	kpn	ST11
155	1775047	m	icu	170722030	BALF	kpn	ST11
156	1775557	f	icu	170807H029	Urine	kpn	ST11
157	1775768	f	icu	1708054030	Urine	kpn	ST11
158	1776183	m	icu	1708091004	Urine	kpn	ST11
159	1776245	f	icu	170721202	Urine	kpn	ST11
160	1776929	f	icu	170801048	Urine	kpn	ST11

161	1777669	m	icu	1708101033	Urine	kpn	ST11
162	1778511	f	icu	170728024	Urine	kpn	ST11
163	1779476	f	icu	170730001	Urine	kpn	ST11
164	1779744	f	icu	170809H060	Urine	kpn	ST11
165	1780136	m	icu	1710014002	Urine	kpn	ST11
166	1780855	m	icu	1708056115	Blood	kpn	ST11
167	1781129	f	icu	1708211061	Blood	kpn	ST11
168	1782896	f	icu	1708301068	Blood	kpn	ST11
169	1783150	m	icu	1709271073	Blood	kpn	ST11
170	1785644	f	icu	1708121081	Blood	kpn	ST11
171	1786611	m	icu	1710181069	Blood	kpn	ST11
172	1786730	m	icu	1708131056	Blood	kpn	ST11
173	1787863	f	icu	1708201002	Blood	kpn	ST11
174	1788964	m	icu	1708261063	Blood	kpn	ST11
175	1789110	f	icu	1708174036	Blood	kpn	ST11
176	1789592	f	icu	1708254024	Blood	kpn	ST11
177	1789827	m	icu	1709251094	Blood	kpn	ST11
178	1789833	m	icu	1709281075	Blood	kpn	ST11
179	1790155	f	icu	1708196105	Blood	kpn	ST15
180	1793330	f	icu	1708291053	Blood	kpn	ST11
181	1795261	f	icu	1709036112	Blood	kpn	ST11
182	1796385	m	icu	170917G002	Blood	kpn	ST11
183	1796449	m	icu	1709171010	Blood	kpn	ST11
184	1798073	f	icu	171021G005	Blood	kpn	ST11
185	1798991	f	icu	1710041049	Blood	kpn	ST11
186	1800441	m	icu	1712261008	Blood	kpn	ST11
187	1800637	m	icu	1709171026	Blood	kpn	ST11
188	1802164	m	icu	1711091112	Blood	kpn	ST11
189	1802353	m	icu	1709251035	Blood	kpn	ST11
190	1803598	f	icu	1709301089	Blood	kpn	ST11
191	1807531	m	icu	1710091076	Blood	kpn	ST722
192	1809155	m	icu	1710191059	Blood	kpn	ST11
193	1809300	m	icu	1710071068	Blood	kpn	ST11
194	1809481	m	icu	1711246082	Blood	kpn	ST11
195	1811703	m	icu	1711164026	Blood	kpn	ST11
196	1812416	m	icu	1710116074	Blood	kpn	ST11
197	1812894	f	icu	1710216107	Blood	kpn	ST11
198	1813076	m	icu	1710134037	Blood	kpn	ST11
199	1815884	m	icu	1710181078	Blood	kpn	ST11
200	1816127	m	icu	1712046024	Blood	kpn	ST11
201	1817365	m	icu	171126G001	Blood	kpn	ST11
202	1819146	m	icu	1711074011	Blood	kpn	ST11
203	1819758	f	icu	1711181086	Blood	kpn	ST11
204	1820477	f	icu	1711041088	Blood	kpn	ST15
205	1821991	f	icu	1711183028	Blood	kpn	ST11
206	1822057	m	icu	1711011009	Blood	kpn	ST1647
207	1822430	m	icu	1711181015	Blood	kpn	ST11
208	1822599	m	icu	1712151124	Blood	kpn	ST11
209	1823146	m	icu	1711041017	Blood	kpn	ST11
210	1824014	m	icu	1711164035	Blood	kpn	ST11
211	1824858	m	icu	1711161088	Blood	kpn	ST11
212	1825262	m	icu	1711271037	Blood	kpn	ST11
213	1825889	m	icu	1711236129	Blood	kpn	ST11
214	1826017	f	icu	1711091105	Blood	kpn	ST11

215	1826067	m	icu	1711261061	Blood	kpn	ST323
216	1826174	m	icu	1711294015	Blood	kpn	ST11
217	1826332	m	icu	1711091097	Pus	kpn	ST11
218	1826389	f	icu	1712151016	Pus	kpn	ST11
219	1826466	m	icu	1712221093	Pus	kpn	ST11
220	1826503	m	icu	1711111081	Pus	kpn	ST11
221	1826507	m	icu	1712091004	Pus	kpn	ST11
222	1826858	f	icu	1711106071	Pus	kpn	ST11
223	1827255	m	icu	1711181020	Pus	kpn	ST323
224	1827473	f	icu	1711131026	Pus	kpn	ST11
225	1827500	m	icu	1711131024	Pus	kpn	ST11
226	1828232	m	icu	1711221010	Pus	kpn	ST11
227	1829427	f	icu	1712041001	Wound secretion	kpn	ST323
228	1829496	m	icu	171127G009	Wound secretion	kpn	ST11
229	1829525	m	icu	1711261083	Wound secretion	kpn	ST1869
230	1830110	f	icu	1712011075	Wound secretion	kpn	ST11
231	1831141	f	icu	1712111077	Wound secretion	kpn	ST11
232	1832787	m	icu	1712171004	Wound secretion	kpn	ST11
233	1833087	f	icu	1711251054	Wound secretion	kpn	ST11
234	1833255	f	icu	1711274022	Wound secretion	kpn	ST11
235	1833931	m	icu	1711264012	Wound secretion	kpn	ST1869
236	1834695	f	icu	1712031031	Wound secretion	kpn	ST11
237	1836111	m	icu	1712091078	Wound secretion	kpn	ST11
238	1836927	m	icu	1712091011	Wound secretion	kpn	ST323
239	1837981	f	icu	1712074046	Wound secretion	kpn	ST11
240	1839209	m	icu	1712131013	Wound secretion	kpn	ST15
241	1843688	f	icu	1712201007	Wound secretion	kpn	ST11
242	1844885	m	icu	1712204018	Wound secretion	kpn	ST11
243	1848366	f	icu	1712281005	Wound secretion	kpn	ST11
244	1848507	m	icu	1712291085	Wound secretion	kpn	ST1869

Table 2							
Number	Patients ID	Piperacillin	Ampicillin-sulbactam	Piperacillin-tazobactam	Ciprofloxacin	Levofloxacin	Cefuroxime
1	1211008	>128	⩾ 32/16	⩾ 128/4	⩾ 1	⩾ 2	⩾ 32
2	1255707	>128	⩾ 32/16	⩾ 128/4	⩾ 1	⩾ 2	⩾ 32
3	1387145	>128	⩾ 32/16	⩾ 128/4	⩾ 1	⩾ 2	⩾ 32
4	1435940	>128	⩾ 32/16	⩾ 128/4	⩾ 1	⩾ 2	⩾ 32
5	1457348	>128	⩾ 32/16	⩾ 128/4	⩾ 1	⩾ 2	⩾ 32
6	1467859	>128	⩾ 32/16	⩾ 128/4	⩾ 1	⩾ 2	⩾ 32
7	1603858	>128	⩾ 32/16	⩾ 128/4	⩾ 1	⩾ 2	⩾ 32
8	1628916	>128	⩾ 32/16	⩾ 128/4	⩾ 1	⩾ 2	⩾ 32
9	1631286	>128	⩾ 32/16	⩾ 128/4	⩾ 1	⩾ 2	⩾ 32
10	1643835	>128	⩾ 32/16	⩾ 128/4	⩾ 1	⩾ 2	⩾ 32
11	1654306	>128	⩾ 32/16	⩾ 128/4	⩽ 0.05	⩽ 0.12	⩾ 32
12	1655141	>128	⩾ 32/16	⩾ 128/4	⩾ 1	⩾ 2	⩾ 32
13	1673544	>128	⩾ 32/16	⩾ 128/4	⩾ 1	⩾ 2	⩾ 32
14	1674016	>128	⩾ 32/16	⩾ 128/4	⩾ 1	⩾ 2	⩾ 32
15	1676693	>128	⩾ 32/16	⩾ 128/4	⩾ 1	⩾ 2	⩾ 32
16	1676967	>128	⩾ 32/16	⩾ 128/4	⩾ 1	⩾ 2	⩾ 32
17	1677363	>128	⩾ 32/16	⩾ 128/4	⩾ 1	⩾ 2	⩾ 32
18	1677521	>128	⩾ 32/16	⩾ 128/4	⩾ 1	⩾ 2	⩾ 32
19	1678002	>128	⩾ 32/16	⩾ 128/4	⩾ 1	⩾ 2	⩾ 32
20	1678087	>128	⩾ 32/16	⩾ 128/4	⩾ 1	⩾ 2	⩽ 4
21	1678329	>128	⩾ 32/16	⩾ 128/4	⩾ 1	⩾ 2	⩾ 32
22	1679510	>128	⩾ 32/16	⩾ 128/4	⩾ 1	⩾ 2	⩾ 32
23	1681169	>128	⩾ 32/16	⩾ 128/4	⩾ 1	⩾ 2	⩾ 32
24	1681287	>128	⩾ 32/16	⩾ 128/4	⩾ 1	⩾ 2	⩾ 32
25	1681407	>128	⩾ 32/16	⩾ 128/4	⩾ 1	⩾ 2	⩾ 32
26	1681610	>128	⩾ 32/16	⩾ 128/4	⩾ 1	⩾ 2	⩾ 32
27	1683217	>128	⩾ 32/16	⩾ 128/4	⩾ 1	⩾ 2	⩾ 32
28	1683428	>128	⩾ 32/16	⩾ 128/4	⩾ 1	⩾ 2	⩾ 32
29	1683853	>128	⩾ 32/16	⩾ 128/4	⩾ 1	⩾ 2	⩾ 32
30	1683893	>128	⩾ 32/16	⩾ 128/4	⩾ 1	⩾ 2	⩾ 32
31	1684044	>128	⩾ 32/16	⩾ 128/4	⩾ 1	⩾ 2	⩾ 32
32	1684190	>128	⩾ 32/16	⩾ 128/4	⩽ 0.05	⩽ 0.12	⩾ 32
33	1684510	>128	⩾ 32/16	⩾ 128/4	⩾ 1	⩾ 2	⩾ 32
34	1684821	>128	⩾ 32/16	⩾ 128/4	⩾ 1	⩾ 2	⩾ 32
35	1685440	>128	⩾ 32/16	⩾ 128/4	⩾ 1	⩾ 2	⩾ 32
36	1687366	>128	⩾ 32/16	⩾ 128/4	⩾ 1	⩾ 2	⩾ 32
37	1688971	>128	⩾ 32/16	⩾ 128/4	⩾ 1	⩾ 2	⩾ 32
38	1689120	>128	⩾ 32/16	⩾ 128/4	⩾ 1	⩾ 2	⩾ 32
39	1689894	>128	⩾ 32/16	⩾ 128/4	⩾ 1	⩾ 2	⩾ 32
40	1690370	>128	⩾ 32/16	⩾ 128/4	⩾ 1	⩾ 2	⩾ 32
41	1691389	>128	⩾ 32/16	⩾ 128/4	⩾ 1	⩾ 2	⩾ 32
42	1692011	>128	⩾ 32/16	⩾ 128/4	⩾ 1	⩾ 2	⩾ 32
43	1695674	>128	⩾ 32/16	⩾ 128/4	⩾ 1	⩾ 2	⩾ 32
44	1696129	>128	⩾ 32/16	⩾ 128/4	⩾ 1	⩾ 2	⩾ 32
45	1696670	>128	⩾ 32/16	⩾ 128/4	⩾ 1	⩾ 2	⩾ 32
46	1698113	>128	⩾ 32/16	⩾ 128/4	⩾ 1	⩾ 2	⩾ 32
47	1700328	>128	⩾ 32/16	⩾ 128/4	⩾ 1	⩾ 2	⩾ 32
48	1700934	>128	⩾ 32/16	⩾ 128/4	⩾ 1	⩾ 2	⩾ 32
49	1702475	>128	⩾ 32/16	⩾ 128/4	⩾ 1	⩾ 2	⩾ 32
50	1703413	>128	⩾ 32/16	⩾ 128/4	⩾ 1	⩾ 2	⩾ 32
51	1704192	>128	⩾ 32/16	⩾ 128/4	⩾ 1	⩾ 2	⩾ 32
52	1706017	>128	⩾ 32/16	⩾ 128/4	⩾ 1	⩾ 2	⩾ 32

53	1707501	>128	≥ 32/16	≥ 128/4	≥ 1	≥ 2	≥ 32
54	1707627	>128	≥ 32/16	≥ 128/4	≥ 1	≥ 2	≥ 32
55	1710844	>128	≥ 32/16	≥ 128/4	≥ 1	≥ 2	≥ 32
56	1711345	>128	≥ 32/16	≥ 128/4	≥ 1	≥ 2	≥ 32
57	1711559	>128	≥ 32/16	≥ 128/4	≥ 1	≥ 2	≥ 32
58	1711905	>128	≥ 32/16	≥ 128/4	≥ 1	≥ 2	≥ 32
59	1712856	>128	≥ 32/16	≥ 128/4	≥ 1	≥ 2	≥ 32
60	1713261	>128	≥ 32/16	≥ 128/4	≥ 1	≥ 2	≥ 32
61	1713952	>128	≥ 32/16	≥ 128/4	≥ 1	≥ 2	≥ 32
62	1714960	>128	≥ 32/16	≥ 128/4	≥ 1	≥ 2	≥ 32
63	1715473	>128	≥ 32/16	≥ 128/4	≥ 1	≥ 2	≥ 32
64	1716196	>128	≥ 32/16	≥ 128/4	≥ 1	≥ 2	≥ 32
65	1717117	>128	≥ 32/16	≥ 128/4	≥ 1	≥ 2	≥ 32
66	1717330	>128	≥ 32/16	≥ 128/4	≥ 1	≥ 2	≥ 32
67	1718209	>128	≥ 32/16	≥ 128/4	≥ 1	≥ 2	≥ 32
68	1718292	>128	≥ 32/16	≥ 128/4	≥ 1	≥ 2	≥ 32
69	1718861	>128	≥ 32/16	≥ 128/4	≥ 1	≥ 2	≥ 32
70	1719002	>128	≥ 32/16	≥ 128/4	≥ 1	≥ 2	≥ 32
71	1719585	>128	≥ 32/16	≥ 128/4	≥ 1	≥ 2	≥ 32
72	1720545	>128	≥ 32/16	≥ 128/4	≥ 1	≥ 2	≥ 32
73	1722263	>128	≥ 32/16	≥ 128/4	≥ 1	≥ 2	≥ 32
74	1722520	>128	≥ 32/16	≥ 128/4	≥ 1	≥ 2	≥ 32
75	1722588	>128	≥ 32/16	≥ 128/4	≥ 1	≥ 2	≥ 32
76	1722653	>128	≥ 32/16	≥ 128/4	≥ 1	≥ 2	≥ 32
77	1722820	>128	≥ 32/16	≥ 128/4	≥ 1	≥ 2	≥ 32
78	1723040	>128	≥ 32/16	≥ 128/4	≥ 1	≥ 2	≥ 32
79	1724993	>128	≥ 32/16	≥ 128/4	≥ 1	≥ 2	≥ 32
80	1725517	>128	≥ 32/16	≥ 128/4	≥ 1	≥ 2	≥ 32
81	1725783	>128	≥ 32/16	≥ 128/4	≥ 1	≤ 0.12	≥ 32
82	1727646	>128	≥ 32/16	≥ 128/4	≥ 1	≥ 2	≥ 32
83	1728409	>128	≥ 32/16	≥ 128/4	≥ 1	≥ 2	≥ 32
84	1728979	>128	≥ 32/16	≥ 128/4	≥ 1	≥ 2	≥ 32
85	1729216	>128	≥ 32/16	≥ 128/4	≥ 1	≥ 2	≥ 32
86	1729494	>128	≥ 32/16	≥ 128/4	≥ 1	≥ 2	≥ 32
87	1732456	>128	≥ 32/16	≥ 128/4	≥ 1	≥ 2	≥ 32
88	1732972	>128	≥ 32/16	≥ 128/4	≥ 1	≥ 2	≥ 32
89	1733175	>128	≥ 32/16	≥ 128/4	≥ 1	≥ 2	≥ 32
90	1733558	>128	≥ 32/16	≥ 128/4	≥ 1	≥ 2	≥ 32
91	1735030	>128	≥ 32/16	≥ 128/4	≥ 1	≥ 2	≥ 32
92	1736316	>128	≥ 32/16	≥ 128/4	≥ 1	≥ 2	≥ 32
93	1736478	>128	≥ 32/16	≥ 128/4	≥ 1	≥ 2	≥ 32
94	1736545	>128	≥ 32/16	≥ 128/4	≤ 0.05	≤ 0.12	≥ 32
95	1736849	>128	≥ 32/16	≥ 128/4	≥ 1	≥ 2	≥ 32
96	1737908	>128	≥ 32/16	≥ 128/4	≥ 1	≥ 2	≥ 32
97	1738572	>128	≥ 32/16	≥ 128/4	≥ 1	≥ 2	≥ 32
98	1738637	>128	≥ 32/16	≥ 128/4	≥ 1	≥ 2	≥ 32
99	1739659	>128	≥ 32/16	≥ 128/4	≥ 1	≥ 2	≥ 32
100	1739709	>128	≥ 32/16	≥ 128/4	≥ 1	≥ 2	≥ 32
101	1740093	>128	≥ 32/16	≥ 128/4	≥ 1	≥ 2	≥ 32
102	1740266	>128	≥ 32/16	≥ 128/4	≥ 1	≥ 2	≥ 32
103	1741070	>128	≥ 32/16	≥ 128/4	≥ 1	≥ 2	≥ 32
104	1746779	>128	≥ 32/16	≥ 128/4	≥ 1	≥ 2	≥ 32
105	1748811	>128	≥ 32/16	≥ 128/4	≥ 1	≥ 2	≥ 32
106	1749608	>128	≥ 32/16	≥ 128/4	≥ 1	≥ 2	≥ 32

107	1749979	>128	≥ 32/16	≥ 128/4	≥ 1	≥ 2	≥ 32
108	1750181	>128	≥ 32/16	≥ 128/4	≥ 1	≥ 2	≥ 32
109	1750600	>128	≥ 32/16	≥ 128/4	≥ 1	≥ 2	≥ 32
110	1750762	>128	≥ 32/16	≥ 128/4	≥ 1	≥ 2	≥ 32
111	1751643	>128	≥ 32/16	≥ 128/4	≥ 1	≥ 2	≥ 32
112	1752526	>128	≥ 32/16	≥ 128/4	≥ 1	≥ 2	≥ 32
113	1753504	>128	≥ 32/16	≥ 128/4	≥ 1	≥ 2	≥ 32
114	1753816	>128	≥ 32/16	≥ 128/4	≥ 1	≥ 2	≥ 32
115	1755284	>128	≥ 32/16	≥ 128/4	≤ 0.05	≤ 0.12	≥ 32
116	1755340	>128	≥ 32/16	≥ 128/4	≥ 1	≥ 2	≥ 32
117	1756051	>128	≥ 32/16	≥ 128/4	≥ 1	≥ 2	≥ 32
118	1756142	>128	≥ 32/16	≥ 128/4	≥ 1	≥ 2	≥ 32
119	1757201	>128	≥ 32/16	≥ 128/4	≥ 1	≥ 2	≥ 32
120	1757736	>128	≥ 32/16	≥ 128/4	≥ 1	≥ 2	≥ 32
121	1758330	>128	≥ 32/16	≥ 128/4	≥ 1	≥ 2	≥ 32
122	1758395	>128	≥ 32/16	≥ 128/4	≥ 1	≥ 2	≥ 32
123	1758448	>128	≥ 32/16	≥ 128/4	≥ 1	≥ 2	≥ 32
124	1759555	>128	≥ 32/16	≥ 128/4	≥ 1	≥ 2	≥ 32
125	1759817	>128	≥ 32/16	≥ 128/4	≤ 0.05	≤ 0.12	≥ 32
126	1760643	>128	≥ 32/16	≥ 128/4	≥ 1	≥ 2	≥ 32
127	1761292	>128	≥ 32/16	≥ 128/4	≥ 1	≥ 2	≥ 32
128	1761352	>128	≥ 32/16	≥ 128/4	≥ 1	≥ 2	≥ 32
129	1762617	>128	≥ 32/16	≥ 128/4	≥ 1	≥ 2	≥ 32
130	1762843	>128	≥ 32/16	≥ 128/4	≥ 1	≥ 2	≥ 32
131	1763053	>128	≥ 32/16	≥ 128/4	≥ 1	≥ 2	≥ 32
132	1763080	>128	≥ 32/16	≥ 128/4	≥ 1	≥ 2	≥ 32
133	1765040	>128	≥ 32/16	≥ 128/4	≥ 1	≥ 2	≥ 32
134	1765508	>128	≥ 32/16	≥ 128/4	≥ 1	≥ 2	≥ 32
135	1765923	>128	≥ 32/16	≥ 128/4	≥ 1	≥ 2	≥ 32
136	1766094	>128	≥ 32/16	≥ 128/4	≥ 1	≥ 2	≥ 32
137	1766123	>128	≥ 32/16	≥ 128/4	≥ 1	≥ 2	≥ 32
138	1766176	>128	≥ 32/16	≥ 128/4	≥ 1	≥ 2	≥ 32
139	1766198	>128	≥ 32/16	≥ 128/4	≥ 1	≥ 2	≥ 32
140	1766489	>128	≥ 32/16	≥ 128/4	≥ 1	≥ 2	≥ 32
141	1767244	>128	≥ 32/16	≥ 128/4	≥ 1	≥ 2	≥ 32
142	1767348	>128	≥ 32/16	≥ 128/4	≥ 1	≥ 2	≥ 32
143	1768100	>128	≥ 32/16	≥ 128/4	≥ 1	≥ 2	≥ 32
144	1769562	>128	≥ 32/16	≥ 128/4	≥ 1	≥ 2	≥ 32
145	1769847	>128	≥ 32/16	≥ 128/4	≥ 1	≥ 2	≥ 32
146	1770111	>128	≥ 32/16	≥ 128/4	≥ 1	≥ 2	≥ 32
147	1770177	>128	≥ 32/16	≥ 128/4	≥ 1	≥ 2	≥ 32
148	1770730	>128	≥ 32/16	≥ 128/4	≥ 1	≥ 2	≥ 32
149	1771549	>128	≥ 32/16	≥ 128/4	≥ 1	≥ 2	≥ 32
150	1772176	>128	≥ 32/16	≥ 128/4	≥ 1	≥ 2	≥ 32
151	1772963	>128	≥ 32/16	≥ 128/4	≥ 1	≥ 2	≥ 32
152	1773213	>128	≥ 32/16	≥ 128/4	≥ 1	≥ 2	≥ 32
153	1773735	>128	≥ 32/16	≥ 128/4	≥ 1	≥ 2	≥ 32
154	1774722	>128	≥ 32/16	≥ 128/4	≥ 1	≥ 2	≥ 32
155	1775047	>128	≥ 32/16	≥ 128/4	≥ 1	≥ 2	≥ 32
156	1775557	>128	≥ 32/16	≥ 128/4	≥ 1	≥ 2	≥ 32
157	1775768	>128	≥ 32/16	≥ 128/4	≥ 1	≥ 2	≥ 32
158	1776183	>128	≥ 32/16	≥ 128/4	≥ 1	≥ 2	≥ 32
159	1776245	>128	≥ 32/16	≥ 128/4	≥ 1	≥ 2	≥ 32
160	1776929	>128	≥ 32/16	≥ 128/4	≥ 1	≥ 2	≥ 32

161	1777669	>128	≥ 32/16	≥ 128/4	≥ 1	≥ 2	≥ 32
162	1778511	>128	≥ 32/16	≥ 128/4	≥ 1	≥ 2	≥ 32
163	1779476	>128	≥ 32/16	≥ 128/4	≥ 1	≥ 2	≥ 32
164	1779744	>128	≥ 32/16	≥ 128/4	≥ 1	≥ 2	≥ 32
165	1780136	>128	≥ 32/16	≥ 128/4	≥ 1	≥ 2	≥ 32
166	1780855	>128	≥ 32/16	≥ 128/4	≥ 1	≥ 2	≥ 32
167	1781129	>128	≥ 32/16	≥ 128/4	≥ 1	≥ 2	≥ 32
168	1782896	>128	≥ 32/16	≥ 128/4	≤ 0.05	≤ 0.12	≥ 32
169	1783150	>128	≥ 32/16	≥ 128/4	≥ 1	≥ 2	≥ 32
170	1785644	>128	≥ 32/16	≥ 128/4	≥ 1	≥ 2	≥ 32
171	1786611	>128	≥ 32/16	≥ 128/4	≥ 1	≥ 2	≥ 32
172	1786730	>128	≥ 32/16	≥ 128/4	≥ 1	≥ 2	≥ 32
173	1787863	>128	≥ 32/16	≥ 128/4	≥ 1	≥ 2	≥ 32
174	1788964	>128	≥ 32/16	≥ 128/4	≥ 1	≥ 2	≥ 32
175	1789110	>128	≥ 32/16	≥ 128/4	≥ 1	≥ 2	≥ 32
176	1789592	>128	≥ 32/16	≥ 128/4	≥ 1	≥ 2	≥ 32
177	1789827	>128	≥ 32/16	≥ 128/4	≥ 1	≥ 2	≥ 32
178	1789833	>128	≥ 32/16	≥ 128/4	≥ 1	≥ 2	≥ 32
179	1790155	>128	≥ 32/16	≥ 128/4	≥ 1	≥ 2	≥ 32
180	1793330	>128	≥ 32/16	≥ 128/4	≥ 1	≥ 2	≥ 32
181	1795261	>128	≥ 32/16	≥ 128/4	≥ 1	≥ 2	≥ 32
182	1796385	>128	≥ 32/16	≥ 128/4	≤ 0.05	≤ 0.12	≥ 32
183	1796449	>128	≥ 32/16	≥ 128/4	≥ 1	≥ 2	≥ 32
184	1798073	>128	≥ 32/16	≥ 128/4	≥ 1	≥ 2	≥ 32
185	1798991	>128	≥ 32/16	≥ 128/4	≥ 1	≥ 2	≥ 32
186	1800441	>128	≥ 32/16	≥ 128/4	≥ 1	≥ 2	≥ 32
187	1800637	>128	≥ 32/16	≥ 128/4	≥ 1	≥ 2	≥ 32
188	1802164	>128	≥ 32/16	≥ 128/4	≥ 1	≥ 2	≥ 32
189	1802353	>128	≥ 32/16	≥ 128/4	≥ 1	≥ 2	≥ 32
190	1803598	>128	≥ 32/16	≥ 128/4	≥ 1	≥ 2	≥ 32
191	1807531	>128	≥ 32/16	≥ 128/4	≥ 1	≥ 2	≥ 32
192	1809155	>128	≥ 32/16	≥ 128/4	≥ 1	≥ 2	≥ 32
193	1809300	>128	≥ 32/16	≥ 128/4	≥ 1	≥ 2	≥ 32
194	1809481	>128	≥ 32/16	≥ 128/4	≥ 1	≥ 2	≥ 32
195	1811703	>128	≥ 32/16	≥ 128/4	≥ 1	≥ 2	≥ 32
196	1812416	>128	≥ 32/16	≥ 128/4	≥ 1	≥ 2	≥ 32
197	1812894	>128	≥ 32/16	≥ 128/4	≥ 1	≥ 2	≥ 32
198	1813076	>128	≥ 32/16	≥ 128/4	≥ 1	≥ 2	≥ 32
199	1815884	>128	≥ 32/16	≥ 128/4	≥ 1	≥ 2	≥ 32
200	1816127	>128	≥ 32/16	≥ 128/4	≥ 1	≥ 2	≥ 32
201	1817365	>128	≥ 32/16	≥ 128/4	≥ 1	≥ 2	≥ 32
202	1819146	>128	≥ 32/16	≥ 128/4	≥ 1	≥ 2	≥ 32
203	1819758	>128	≥ 32/16	≥ 128/4	≥ 1	≥ 2	≥ 32
204	1820477	>128	≥ 32/16	≥ 128/4	≥ 1	≥ 2	≥ 32
205	1821991	>128	≥ 32/16	≥ 128/4	≥ 1	≥ 2	≥ 32
206	1822057	>128	≥ 32/16	≥ 128/4	≥ 1	≥ 2	≥ 32
207	1822430	>128	≥ 32/16	≥ 128/4	≥ 1	≥ 2	≥ 32
208	1822599	>128	≥ 32/16	≥ 128/4	≥ 1	≥ 2	≥ 32
209	1823146	>128	≥ 32/16	≥ 128/4	≥ 1	≥ 2	≥ 32
210	1824014	>128	≥ 32/16	≥ 128/4	≥ 1	≥ 2	≥ 32
211	1824858	>128	≥ 32/16	≥ 128/4	≥ 1	≥ 2	≥ 32
212	1825262	>128	≥ 32/16	≥ 128/4	≥ 1	≥ 2	≥ 32
213	1825889	>128	≥ 32/16	≥ 128/4	≥ 1	≥ 2	≥ 32
214	1826017	>128	≥ 32/16	≥ 128/4	≥ 1	≥ 2	≥ 32

215	1826067	>128	≥ 32/16	≥ 128/4	≥ 1	≥ 2	≥ 32
216	1826174	>128	≥ 32/16	≥ 128/4	≥ 1	≥ 2	≥ 32
217	1826332	>128	≥ 32/16	≥ 128/4	≥ 1	≥ 2	≥ 32
218	1826389	>128	≥ 32/16	≥ 128/4	≥ 1	≥ 2	≥ 32
219	1826466	>128	≥ 32/16	≥ 128/4	≥ 1	≥ 2	≥ 32
220	1826503	>128	≥ 32/16	≥ 128/4	≥ 1	≥ 2	≥ 32
221	1826507	>128	≥ 32/16	≥ 128/4	≥ 1	≥ 2	≥ 32
222	1826858	>128	≥ 32/16	≥ 128/4	≥ 1	≥ 2	≥ 32
223	1827255	>128	≥ 32/16	≥ 128/4	≥ 1	≥ 2	≥ 32
224	1827473	>128	≥ 32/16	≥ 128/4	≥ 1	≥ 2	≥ 32
225	1827500	>128	≥ 32/16	≥ 128/4	≥ 1	≤ 0.12	≥ 32
226	1828232	>128	≥ 32/16	≥ 128/4	≥ 1	≥ 2	≥ 32
227	1829427	>128	≥ 32/16	≥ 128/4	≥ 1	≥ 2	≥ 32
228	1829496	>128	≥ 32/16	≥ 128/4	≥ 1	≥ 2	≥ 32
229	1829525	>128	≥ 32/16	≥ 128/4	≥ 1	≥ 2	≥ 32
230	1830110	>128	≥ 32/16	≥ 128/4	≥ 1	≥ 2	≥ 32
231	1831141	>128	≥ 32/16	≥ 128/4	≥ 1	≥ 2	≥ 32
232	1832787	>128	≥ 32/16	≥ 128/4	≥ 1	≥ 2	≥ 32
233	1833087	>128	≥ 32/16	≥ 128/4	≥ 1	≥ 2	≥ 32
234	1833255	>128	≥ 32/16	≥ 128/4	≥ 1	≥ 2	≥ 32
235	1833931	>128	≥ 32/16	≥ 128/4	≥ 1	≥ 2	≥ 32
236	1834695	>128	≥ 32/16	≥ 128/4	≥ 1	≥ 2	≥ 32
237	1836111	>128	≥ 32/16	≥ 128/4	≥ 1	≥ 2	≥ 32
238	1836927	>128	≥ 32/16	≥ 128/4	≥ 1	≥ 2	≥ 32
239	1837981	>128	≥ 32/16	≥ 128/4	≥ 1	≥ 2	≥ 32
240	1839209	>128	≥ 32/16	≥ 128/4	≥ 1	≥ 2	≥ 32
241	1843688	>128	≥ 32/16	≥ 128/4	≥ 1	≥ 2	≥ 32
242	1844885	>128	≥ 32/16	≥ 128/4	≥ 1	≥ 2	≥ 32
243	1848366	>128	≥ 32/16	≥ 128/4	≥ 1	≥ 2	≥ 32
244	1848507	>128	≥ 32/16	≥ 128/4	≥ 1	≥ 2	≥ 32

Table 3									
Number	Patients ID	Ceftazidime	Cefepime	Aztreonam	Amikacin	Gentamicin	Fosfomycin	Tigecycline	Trimethoprim-sulfamethoxazole
1	1211008	≥ 16	≥ 16	≥ 16	≥ 64	≥ 16	≥ 256	≤ 1	≥4/76
2	1255707	≥ 16	≥ 16	≥ 16	≤ 16	≤ 4	≥ 256	≤ 1	≥4/76
3	1387145	≥ 16	≥ 16	≥ 16	≥ 64	≥ 16	≤ 64	≤ 1	≥4/76
4	1435940	≥ 16	≥ 16	≥ 16	≥ 64	≥ 16	≥ 256	≤ 1	≥4/76
5	1457348	≥ 16	≥ 16	≥ 16	≥ 64	≥ 16	≥ 256	≤ 1	≥4/76
6	1467859	≥ 16	≥ 16	≥ 16	≥ 64	≥ 16	≤ 64	≤ 1	≥4/76
7	1603858	≥ 16	≥ 16	≥ 16	≥ 64	≥ 16	≥ 256	≤ 1	≥4/76
8	1628916	≥ 16	≥ 16	≥ 16	≥ 64	≥ 16	≥ 256	≤ 1	≥4/76
9	1631286	≥ 16	≥ 16	≥ 16	≥ 64	≥ 16	≤ 64	≤ 1	≥4/76
10	1643835	≥ 16	≥ 16	≥ 16	≥ 64	≥ 16	≥ 256	≤ 1	≥4/76
11	1654306	≤ 4	≤ 2	≥ 16	≤ 16	≤ 4	≥ 256	≥ 4	≥4/76
12	1655141	≥ 16	≥ 16	≥ 16	≥ 64	≥ 16	≤ 64	≤ 1	≥4/76
13	1673544	≥ 16	≥ 16	≥ 16	≥ 64	≥ 16	≥ 256	≤ 1	≥4/76
14	1674016	≥ 16	≥ 16	≥ 16	≥ 64	≥ 16	≥ 256	≤ 1	≥4/76
15	1676693	≥ 16	≥ 16	≥ 16	≥ 64	≥ 16	≥ 256	≤ 1	≥4/76
16	1676967	≥ 16	≥ 16	≥ 16	≥ 64	≥ 16	≤ 64	≤ 1	≥4/76
17	1677363	≥ 16	≥ 16	≥ 16	≥ 64	≥ 16	≥ 256	≤ 1	≥4/76
18	1677521	≥ 16	≥ 16	≥ 16	≥ 64	≥ 16	≥ 256	≤ 1	≥4/76
19	1678002	≥ 16	≥ 16	≥ 16	≤ 16	≥ 16	≤ 64	≥ 4	≥4/76
20	1678087	≥ 16	≥ 16	≥ 16	≤ 16	≤ 4	≥ 256	≤ 1	≥4/76
21	1678329	≥ 16	≥ 16	≥ 16	≥ 64	≥ 16	≥ 256	≤ 1	≥4/76
22	1679510	≥ 16	≥ 16	≥ 16	≥ 64	≥ 16	≤ 64	≤ 1	≥4/76
23	1681169	≥ 16	≥ 16	≥ 16	≥ 64	≥ 16	≥ 256	≤ 1	≥4/76
24	1681287	≥ 16	≥ 16	≥ 16	≥ 64	≥ 16	≥ 256	≥ 4	≥4/76
25	1681407	≥ 16	≥ 16	≥ 16	≤ 16	≤ 4	≥ 256	≤ 1	≥4/76
26	1681610	≥ 16	≥ 16	≥ 16	≥ 64	≥ 16	≤ 64	≤ 1	≥4/76
27	1683217	≥ 16	≥ 16	≥ 16	≥ 64	≥ 16	≤ 64	≤ 1	≥4/76
28	1683428	≥ 16	≥ 16	≥ 16	≥ 64	≥ 16	≥ 256	≤ 1	≥4/76
29	1683853	≥ 16	≥ 16	≥ 16	≥ 64	≥ 16	≥ 256	≤ 1	≥4/76
30	1683893	≥ 16	≥ 16	≥ 16	≥ 64	≥ 16	≥ 256	≤ 1	≥4/76
31	1684044	≥ 16	≥ 16	≥ 16	≥ 64	≥ 16	≤ 64	≤ 1	≥4/76
32	1684190	≥ 16	≥ 16	≥ 16	≤ 16	≥ 16	≥ 256	≤ 1	≥4/76
33	1684510	≥ 16	≥ 16	≥ 16	≥ 64	≥ 16	≥ 256	≤ 1	≥4/76
34	1684821	≥ 16	≥ 16	≥ 16	≤ 16	≥ 16	≤ 64	≤ 1	≥4/76
35	1685440	≥ 16	≥ 16	≥ 16	≥ 64	≥ 16	≥ 256	≤ 1	≥4/76
36	1687366	≥ 16	≥ 16	≥ 16	≥ 64	≥ 16	≤ 64	≤ 1	≥4/76
37	1688971	≤ 4	≤ 2	≥ 16	≥ 64	≥ 16	≥ 256	≤ 1	≥4/76
38	1689120	≥ 16	≥ 16	≥ 16	≥ 64	≥ 16	≥ 256	≤ 1	≥4/76
39	1689894	≥ 16	≥ 16	≥ 16	≤ 16	≥ 16	≥ 256	≤ 1	≥4/76
40	1690370	≥ 16	≥ 16	≥ 16	≥ 64	≥ 16	≥ 256	≤ 1	≥4/76
41	1691389	≥ 16	≥ 16	≥ 16	≥ 64	≥ 16	≤ 64	≤ 1	≥4/76
42	1692011	≥ 16	≥ 16	≥ 16	≥ 64	≥ 16	≥ 256	≤ 1	≥4/76
43	1695674	≥ 16	≥ 16	≥ 16	≥ 64	≥ 16	≥ 256	≤ 1	≥4/76
44	1696129	≥ 16	≥ 16	≥ 16	≤ 16	≥ 16	≤ 64	≤ 1	≥4/76
45	1696670	≥ 16	≥ 16	≥ 16	≥ 64	≥ 16	≥ 256	≥ 4	≥4/76
46	1698113	≥ 16	≥ 16	≥ 16	≥ 64	≥ 16	≥ 256	≤ 1	≥4/76
47	1700328	≥ 16	≥ 16	≥ 16	≥ 64	≥ 16	≥ 256	≤ 1	≥4/76
48	1700934	≥ 16	≥ 16	≥ 16	≥ 64	≥ 16	≤ 64	≤ 1	≥4/76
49	1702475	≥ 16	≥ 16	≥ 16	≤ 16	≥ 16	≥ 256	≤ 1	≥4/76
50	1703413	≥ 16	≥ 16	≥ 16	≥ 64	≥ 16	≥ 256	≤ 1	≥4/76
51	1704192	≥ 16	≥ 16	≥ 16	≥ 64	≥ 16	≥ 256	≤ 1	≥4/76
52	1706017	≥ 16	≥ 16	≥ 16	≥ 64	≥ 16	≤ 64	≤ 1	≥4/76

53	1707501	≥ 16	≥ 16	≥ 16	≤ 16	≥ 16	≥ 256	≤ 1	≥4/76
54	1707627	≥ 16	≥ 16	≥ 16	≥ 64	≥ 16	≥ 256	≤ 1	≥4/76
55	1710844	≥ 16	≥ 16	≥ 16	≥ 64	≥ 16	≥ 256	≤ 1	≥4/76
56	1711345	≥ 16	≥ 16	≥ 16	≥ 64	≥ 16	≥ 256	≤ 1	≥4/76
57	1711559	≥ 16	≥ 16	≥ 16	≥ 64	≥ 16	≤ 64	≤ 1	≥4/76
58	1711905	≥ 16	≥ 16	≥ 16	≤ 16	≥ 16	≥ 256	≤ 1	≥4/76
59	1712856	≥ 16	≥ 16	≥ 16	≥ 64	≥ 16	≥ 256	≤ 1	≥4/76
60	1713261	≥ 16	≥ 16	≥ 16	≥ 64	≥ 16	≤ 64	≤ 1	≥4/76
61	1713952	≥ 16	≥ 16	≥ 16	≥ 64	≥ 16	≤ 64	≤ 1	≥4/76
62	1714960	≥ 16	≥ 16	≥ 16	≤ 16	≥ 16	≥ 256	≤ 1	≥4/76
63	1715473	≥ 16	≥ 16	≥ 16	≥ 64	≥ 16	≥ 256	≥ 4	≥4/76
64	1716196	≥ 16	≥ 16	≥ 16	≥ 64	≥ 16	≤ 64	≤ 1	≥4/76
65	1717117	≥ 16	≥ 16	≥ 16	≥ 64	≥ 16	≥ 256	≤ 1	≥4/76
66	1717330	≥ 16	≥ 16	≥ 16	≥ 64	≥ 16	≥ 256	≤ 1	≥4/76
67	1718209	≥ 16	≥ 16	≥ 16	≥ 64	≥ 16	≤ 64	≤ 1	≥4/76
68	1718292	≥ 16	≥ 16	≥ 16	≥ 64	≥ 16	≥ 256	≤ 1	≥4/76
69	1718861	≥ 16	≥ 16	≥ 16	≥ 64	≥ 16	≥ 256	≤ 1	≥4/76
70	1719002	≥ 16	≥ 16	≥ 16	≤ 16	≥ 16	≥ 256	≤ 1	≥4/76
71	1719585	≥ 16	≥ 16	≥ 16	≥ 64	≥ 16	≥ 256	≤ 1	≥4/76
72	1720545	≥ 16	≥ 16	≥ 16	≥ 64	≥ 16	≤ 64	≤ 1	≥4/76
73	1722263	≥ 16	≥ 16	≥ 16	≥ 64	≥ 16	≥ 256	≤ 1	≥4/76
74	1722520	≥ 16	≥ 16	≥ 16	≥ 64	≥ 16	≥ 256	≤ 1	≥4/76
75	1722588	≥ 16	≥ 16	≥ 16	≥ 64	≥ 16	≥ 256	≤ 1	≥4/76
76	1722653	≥ 16	≥ 16	≥ 16	≥ 64	≥ 16	≥ 256	≤ 1	≥4/76
77	1722820	≥ 16	≥ 16	≥ 16	≥ 64	≥ 16	≤ 64	≤ 1	≥4/76
78	1723040	≥ 16	≥ 16	≥ 16	≥ 64	≥ 16	≥ 256	≤ 1	≥4/76
79	1724993	≥ 16	≥ 16	≥ 16	≤ 16	≥ 16	≤ 64	≤ 1	≥4/76
80	1725517	≥ 16	≥ 16	≥ 16	≥ 64	≥ 16	≥ 256	≤ 1	≥4/76
81	1725783	≥ 16	≥ 16	≥ 16	≤ 16	≥ 16	≤ 64	≥ 4	≥4/76
82	1727646	≥ 16	≥ 16	≥ 16	≥ 64	≥ 16	≥ 256	≤ 1	≥4/76
83	1728409	≥ 16	≥ 16	≥ 16	≥ 64	≥ 16	≤ 64	≤ 1	≥4/76
84	1728979	≥ 16	≥ 16	≥ 16	≤ 16	≥ 16	≥ 256	≤ 1	≥4/76
85	1729216	≥ 16	≥ 16	≥ 16	≥ 64	≥ 16	≥ 256	≤ 1	≥4/76
86	1729494	≥ 16	≥ 16	≥ 16	≥ 64	≥ 16	≥ 256	≤ 1	≥4/76
87	1732456	≥ 16	≥ 16	≥ 16	≤ 16	≥ 16	≤ 64	≤ 1	≥4/76
88	1732972	≥ 16	≥ 16	≥ 16	≥ 64	≥ 16	≥ 256	≤ 1	≥4/76
89	1733175	≥ 16	≥ 16	≥ 16	≤ 16	≥ 16	≤ 64	≤ 1	≥4/76
90	1733558	≥ 16	≥ 16	≥ 16	≥ 64	≥ 16	≥ 256	≤ 1	≥4/76
91	1735030	≥ 16	≥ 16	≥ 16	≤ 16	≥ 16	≥ 256	≤ 1	≥4/76
92	1736316	≥ 16	≥ 16	≥ 16	≤ 16	≥ 16	≤ 64	≤ 1	≥4/76
93	1736478	≥ 16	≥ 16	≥ 16	≥ 64	≥ 16	≥ 256	≤ 1	≥4/76
94	1736545	≥ 16	≥ 16	≥ 16	≤ 16	≥ 16	≥ 256	≤ 1	≥4/76
95	1736849	≥ 16	≥ 16	≥ 16	≥ 64	≥ 16	≤ 64	≤ 1	≥4/76
96	1737908	≥ 16	≥ 16	≥ 16	≤ 16	≥ 16	≥ 256	≤ 1	≥4/76
97	1738572	≥ 16	≥ 16	≥ 16	≥ 64	≥ 16	≥ 256	≤ 1	≥4/76
98	1738637	≥ 16	≥ 16	≥ 16	≤ 16	≥ 16	≤ 64	≥ 4	≥4/76
99	1739659	≥ 16	≥ 16	≥ 16	≥ 64	≥ 16	≤ 64	≤ 1	≥4/76
100	1739709	≥ 16	≥ 16	≥ 16	≤ 16	≥ 16	≥ 256	≤ 1	≥4/76
101	1740093	≥ 16	≥ 16	≥ 16	≥ 64	≥ 16	≤ 64	≤ 1	≥4/76
102	1740266	≥ 16	≥ 16	≥ 16	≤ 16	≥ 16	≥ 256	≤ 1	≥4/76
103	1741070	≥ 16	≥ 16	≥ 16	≥ 64	≥ 16	≥ 256	≤ 1	≥4/76
104	1746779	≤ 4	≤ 2	≥ 16	≤ 16	≥ 16	≤ 64	≤ 1	≥4/76
105	1748811	≥ 16	≥ 16	≥ 16	≤ 16	≥ 16	≥ 256	≤ 1	≥4/76
106	1749608	≥ 16	≥ 16	≥ 16	≥ 64	≥ 16	≤ 64	≤ 1	≥4/76

107	1749979	≥ 16	≥ 16	≥ 16	≤ 16	≤ 4	≥ 256	≤ 1	≥4/76
108	1750181	≥ 16	≥ 16	≥ 16	≥ 64	≥ 16	≤ 64	≤ 1	≥4/76
109	1750600	≥ 16	≥ 16	≥ 16	≤ 16	≤ 4	≥ 256	≤ 1	≥4/76
110	1750762	≥ 16	≥ 16	≥ 16	≥ 64	≥ 16	≥ 256	≤ 1	≥4/76
111	1751643	≥ 16	≥ 16	≥ 16	≥ 64	≥ 16	≤ 64	≤ 1	≥4/76
112	1752526	≥ 16	≥ 16	≥ 16	≤ 16	≥ 16	≤ 64	≤ 1	≥4/76
113	1753504	≥ 16	≥ 16	≥ 16	≥ 64	≥ 16	≥ 256	≤ 1	≥4/76
114	1753816	≥ 16	≥ 16	≥ 16	≤ 16	≥ 16	≤ 64	≥ 4	≥4/76
115	1755284	≥ 16	≥ 16	≥ 16	≤ 16	≥ 16	≤ 64	≤ 1	≥4/76
116	1755340	≥ 16	≥ 16	≥ 16	≥ 64	≥ 16	≥ 256	≤ 1	≥4/76
117	1756051	≥ 16	≥ 16	≥ 16	≤ 16	≥ 16	≥ 256	≤ 1	≥4/76
118	1756142	≥ 16	≥ 16	≥ 16	≥ 64	≥ 16	≤ 64	≤ 1	≥4/76
119	1757201	≥ 16	≥ 16	≥ 16	≤ 16	≥ 16	≥ 256	≤ 1	≥4/76
120	1757736	≥ 16	≥ 16	≥ 16	≤ 16	≥ 16	≥ 256	≤ 1	≥4/76
121	1758330	≥ 16	≥ 16	≥ 16	≥ 64	≥ 16	≤ 64	≤ 1	≥4/76
122	1758395	≥ 16	≥ 16	≥ 16	≤ 16	≥ 16	≤ 64	≤ 1	≥4/76
123	1758448	≥ 16	≥ 16	≥ 16	≥ 64	≥ 16	≥ 256	≤ 1	≥4/76
124	1759555	≥ 16	≥ 16	≥ 16	≥ 64	≥ 16	≥ 256	≤ 1	≥4/76
125	1759817	≥ 16	≤ 2	≥ 16	≤ 16	≥ 16	≤ 64	≤ 1	≥4/76
126	1760643	≥ 16	≥ 16	≥ 16	≥ 64	≥ 16	≥ 256	≥ 4	≥4/76
127	1761292	≥ 16	≥ 16	≥ 16	≤ 16	≥ 16	≥ 256	≤ 1	≥4/76
128	1761352	≥ 16	≥ 16	≥ 16	≥ 64	≥ 16	≤ 64	≤ 1	≥4/76
129	1762617	≥ 16	≥ 16	≥ 16	≤ 16	≥ 16	≥ 256	≤ 1	≥4/76
130	1762843	≥ 16	≥ 16	≥ 16	≥ 64	≥ 16	≥ 256	≤ 1	≥4/76
131	1763053	≥ 16	≥ 16	≥ 16	≤ 16	≥ 16	≤ 64	≤ 1	≥4/76
132	1763080	≥ 16	≥ 16	≥ 16	≥ 64	≥ 16	≥ 256	≤ 1	≥4/76
133	1765040	≥ 16	≥ 16	≥ 16	≥ 64	≥ 16	≤ 64	≤ 1	≥4/76
134	1765508	≥ 16	≥ 16	≥ 16	≤ 16	≥ 16	≤ 64	≤ 1	≥4/76
135	1765923	≥ 16	≥ 16	≥ 16	≥ 64	≥ 16	≥ 256	≤ 1	≥4/76
136	1766094	≥ 16	≥ 16	≥ 16	≤ 16	≥ 16	≥ 256	≤ 1	≥4/76
137	1766123	≥ 16	≥ 16	≥ 16	≥ 64	≥ 16	≤ 64	≤ 1	≥4/76
138	1766176	≥ 16	≥ 16	≥ 16	≤ 16	≥ 16	≥ 256	≤ 1	≥4/76
139	1766198	≥ 16	≥ 16	≥ 16	≤ 16	≥ 16	≥ 256	≥ 4	≥4/76
140	1766489	≥ 16	≥ 16	≥ 16	≤ 16	≥ 16	≤ 64	≤ 1	≥4/76
141	1767244	≥ 16	≥ 16	≥ 16	≥ 64	≥ 16	≤ 64	≤ 1	≥4/76
142	1767348	≥ 16	≥ 16	≥ 16	≤ 16	≤ 4	≥ 256	≤ 1	≥4/76
143	1768100	≥ 16	≥ 16	≥ 16	≥ 64	≥ 16	≤ 64	≤ 1	≥4/76
144	1769562	≥ 16	≥ 16	≥ 16	≤ 16	≤ 4	≥ 256	≤ 1	≥4/76
145	1769847	≥ 16	≥ 16	≥ 16	≥ 64	≥ 16	≥ 256	≤ 1	≥4/76
146	1770111	≥ 16	≥ 16	≥ 16	≥ 64	≥ 16	≤ 64	≤ 1	≥4/76
147	1770177	≥ 16	≥ 16	≥ 16	≤ 16	≥ 16	≥ 256	≤ 1	≥4/76
148	1770730	≥ 16	≥ 16	≥ 16	≥ 64	≥ 16	≥ 256	≤ 1	≥4/76
149	1771549	≥ 16	≥ 16	≥ 16	≤ 16	≥ 16	≤ 64	≤ 1	≥4/76
150	1772176	≥ 16	≥ 16	≥ 16	≤ 16	≥ 16	≥ 256	≤ 1	≥4/76
151	1772963	≥ 16	≥ 16	≥ 16	≥ 64	≥ 16	≤ 64	≤ 1	≥4/76
152	1773213	≥ 16	≥ 16	≥ 16	≥ 64	≥ 16	≥ 256	≥ 4	≥4/76
153	1773735	≥ 16	≥ 16	≥ 16	≥ 64	≥ 16	≤ 64	≤ 1	≥4/76
154	1774722	≥ 16	≥ 16	≥ 16	≤ 16	≤ 4	≥ 256	≤ 1	≥4/76
155	1775047	≥ 16	≥ 16	≥ 16	≥ 64	≤ 4	≤ 64	≤ 1	≥4/76
156	1775557	≥ 16	≥ 16	≥ 16	≤ 16	≤ 4	≥ 256	≤ 1	≥4/76
157	1775768	≥ 16	≥ 16	≥ 16	≥ 64	≥ 16	≥ 256	≤ 1	≥4/76
158	1776183	≥ 16	≥ 16	≥ 16	≥ 64	≥ 16	≥ 256	≤ 1	≥4/76
159	1776245	≥ 16	≥ 16	≥ 16	≤ 16	≤ 4	≥ 256	≤ 1	≥4/76
160	1776929	≥ 16	≥ 16	≥ 16	≥ 64	≥ 16	≤ 64	≤ 1	≥4/76

161	1777669	≥ 16	≥ 16	≥ 16	≥ 64	≥ 16	≥ 256	≤ 1	≥4/76
162	1778511	≥ 16	≥ 16	≥ 16	≤ 16	≥ 16	≥ 256	≤ 1	≥4/76
163	1779476	≥ 16	≥ 16	≥ 16	≤ 16	≤ 4	≤ 64	≤ 1	≥4/76
164	1779744	≥ 16	≥ 16	≥ 16	≥ 64	≥ 16	≥ 256	≤ 1	≥4/76
165	1780136	≥ 16	≥ 16	≥ 16	≥ 64	≥ 16	≥ 256	≤ 1	≥4/76
166	1780855	≥ 16	≥ 16	≥ 16	≤ 16	≥ 16	≥ 256	≤ 1	≥4/76
167	1781129	≥ 16	≥ 16	≥ 16	≥ 64	≥ 16	≥ 256	≤ 1	≥4/76
168	1782896	≥ 16	≥ 16	≥ 16	≥ 64	≥ 16	≥ 256	≤ 1	≥4/76
169	1783150	≥ 16	≥ 16	≥ 16	≤ 16	≤ 4	≥ 256	≤ 1	≥4/76
170	1785644	≥ 16	≥ 16	≥ 16	≥ 64	≥ 16	≥ 256	≤ 1	≥4/76
171	1786611	≥ 16	≥ 16	≥ 16	≤ 16	≥ 16	≥ 256	≤ 1	≥4/76
172	1786730	≥ 16	≥ 16	≥ 16	≥ 64	≥ 16	≥ 256	≤ 1	≥4/76
173	1787863	≥ 16	≥ 16	≥ 16	≥ 64	≥ 16	≥ 256	≤ 1	≥4/76
174	1788964	≥ 16	≥ 16	≥ 16	≥ 64	≥ 16	≥ 256	≤ 1	≥4/76
175	1789110	≥ 16	≥ 16	≥ 16	≤ 16	≤ 4	≤ 64	≤ 1	≥4/76
176	1789592	≥ 16	≥ 16	≥ 16	≥ 64	≥ 16	≥ 256	≤ 1	≥4/76
177	1789827	≥ 16	≥ 16	≥ 16	≤ 16	≤ 4	≤ 64	≤ 1	≥4/76
178	1789833	≥ 16	≥ 16	≥ 16	≥ 64	≥ 16	≥ 256	≤ 1	≥4/76
179	1790155	≥ 16	≥ 16	≥ 16	≤ 16	≤ 4	≥ 256	≤ 1	≥4/76
180	1793330	≥ 16	≥ 16	≥ 16	≥ 64	≥ 16	≥ 256	≤ 1	≥4/76
181	1795261	≥ 16	≥ 16	≥ 16	≥ 64	≥ 16	≥ 256	≤ 1	≥4/76
182	1796385	≥ 16	≥ 16	≥ 16	≥ 64	≥ 16	≥ 256	≤ 1	≥4/76
183	1796449	≥ 16	≥ 16	≥ 16	≤ 16	≥ 16	≥ 256	≤ 1	≥4/76
184	1798073	≥ 16	≥ 16	≥ 16	≥ 64	≥ 16	≤ 64	≤ 1	≥4/76
185	1798991	≥ 16	≥ 16	≥ 16	≥ 64	≥ 16	≥ 256	≤ 1	≥4/76
186	1800441	≥ 16	≥ 16	≥ 16	≤ 16	≤ 4	≥ 256	≤ 1	≥4/76
187	1800637	≥ 16	≥ 16	≥ 16	≥ 64	≥ 16	≤ 64	≤ 1	≥4/76
188	1802164	≥ 16	≥ 16	≥ 16	≤ 16	≥ 16	≥ 256	≤ 1	≥4/76
189	1802353	≥ 16	≥ 16	≥ 16	≥ 64	≥ 16	≤ 64	≤ 1	≥4/76
190	1803598	≥ 16	≥ 16	≥ 16	≤ 16	≤ 4	≥ 256	≤ 1	≥4/76
191	1807531	≥ 16	≥ 16	≥ 16	≤ 16	≥ 16	≤ 64	≤ 1	≥4/76
192	1809155	≥ 16	≥ 16	≥ 16	≥ 64	≥ 16	≥ 256	≤ 1	≥4/76
193	1809300	≥ 16	≥ 16	≥ 16	≤ 16	≤ 4	≥ 256	≤ 1	≥4/76
194	1809481	≥ 16	≥ 16	≥ 16	≥ 64	≥ 16	≤ 64	≤ 1	≥4/76
195	1811703	≥ 16	≥ 16	≥ 16	≤ 16	≤ 4	≥ 256	≤ 1	≥4/76
196	1812416	≥ 16	≥ 16	≥ 16	≥ 64	≥ 16	≤ 64	≤ 1	≥4/76
197	1812894	≥ 16	≥ 16	≥ 16	≤ 16	≤ 4	≤ 64	≤ 1	≥4/76
198	1813076	≥ 16	≥ 16	≥ 16	≥ 64	≥ 16	≥ 256	≤ 1	≥4/76
199	1815884	≥ 16	≥ 16	≥ 16	≥ 64	≥ 16	≥ 256	≤ 1	≥4/76
200	1816127	≥ 16	≥ 16	≥ 16	≥ 64	≥ 16	≥ 256	≤ 1	≥4/76
201	1817365	≥ 16	≥ 16	≥ 16	≥ 64	≥ 16	≥ 256	≤ 1	≥4/76
202	1819146	≥ 16	≥ 16	≥ 16	≥ 64	≥ 16	≤ 64	≤ 1	≥4/76
203	1819758	≥ 16	≥ 16	≥ 16	≤ 16	≤ 4	≥ 256	≤ 1	≥4/76
204	1820477	≥ 16	≥ 16	≥ 16	≥ 64	≥ 16	≤ 64	≤ 1	≥4/76
205	1821991	≥ 16	≥ 16	≥ 16	≤ 16	≤ 4	≤ 64	≤ 1	≥4/76
206	1822057	≥ 16	≥ 16	≥ 16	≥ 64	≥ 16	≥ 256	≤ 1	≥4/76
207	1822430	≥ 16	≥ 16	≥ 16	≥ 64	≥ 16	≤ 64	≤ 1	≥4/76
208	1822599	≥ 16	≥ 16	≥ 16	≥ 64	≥ 16	≥ 256	≤ 1	≥4/76
209	1823146	≥ 16	≥ 16	≥ 16	≤ 16	≤ 4	≤ 64	≤ 1	≥4/76
210	1824014	≥ 16	≥ 16	≥ 16	≤ 16	≤ 4	≥ 256	≤ 1	≥4/76
211	1824858	≥ 16	≥ 16	≥ 16	≥ 64	≥ 16	≥ 256	≤ 1	≥4/76
212	1825262	≥ 16	≥ 16	≥ 16	≥ 64	≥ 16	≥ 256	≤ 1	≥4/76
213	1825889	≥ 16	≥ 16	≥ 16	≤ 16	≤ 4	≤ 64	≤ 1	≥4/76
214	1826017	≥ 16	≥ 16	≥ 16	≥ 64	≥ 16	≥ 256	≤ 1	≥4/76

215	1826067	≥ 16	≥ 16	≥ 16	≥ 64	≥ 16	≥ 256	≤ 1	≥4/76
216	1826174	≥ 16	≥ 16	≥ 16	≤ 16	≤ 4	≤ 64	≤ 1	≥4/76
217	1826332	≥ 16	≥ 16	≥ 16	≥ 64	≥ 16	≥ 256	≤ 1	≥4/76
218	1826389	≥ 16	≥ 16	≥ 16	≥ 64	≥ 16	≤ 64	≤ 1	≥4/76
219	1826466	≥ 16	≥ 16	≥ 16	≥ 64	≥ 16	≥ 256	≤ 1	≥4/76
220	1826503	≥ 16	≥ 16	≥ 16	≥ 64	≥ 16	≥ 256	≤ 1	≥4/76
221	1826507	≥ 16	≥ 16	≥ 16	≥ 64	≥ 16	≥ 256	≤ 1	≥4/76
222	1826858	≥ 16	≥ 16	≥ 16	≥ 64	≥ 16	≤ 64	≤ 1	≥4/76
223	1827255	≥ 16	≥ 16	≥ 16	≥ 64	≥ 16	≥ 256	≤ 1	≥4/76
224	1827473	≥ 16	≥ 16	≥ 16	≥ 64	≥ 16	≥ 256	≤ 1	≥4/76
225	1827500	≥ 16	≥ 16	≥ 16	≥ 64	≥ 16	≤ 64	≤ 1	≥4/76
226	1828232	≥ 16	≥ 16	≥ 16	≥ 64	≥ 16	≥ 256	≤ 1	≥4/76
227	1829427	≥ 16	≥ 16	≥ 16	≥ 64	≥ 16	≤ 64	≤ 1	≥4/76
228	1829496	≥ 16	≥ 16	≥ 16	≥ 64	≥ 16	≥ 256	≤ 1	≥4/76
229	1829525	≥ 16	≥ 16	≥ 16	≥ 64	≥ 16	≤ 64	≤ 1	≥4/76
230	1830110	≥ 16	≥ 16	≥ 16	≥ 64	≥ 16	≥ 256	≤ 1	≥4/76
231	1831141	≥ 16	≥ 16	≥ 16	≥ 64	≥ 16	≤ 64	≤ 1	≥4/76
232	1832787	≥ 16	≥ 16	≥ 16	≥ 64	≥ 16	≥ 256	≤ 1	≥4/76
233	1833087	≥ 16	≥ 16	≥ 16	≥ 64	≥ 16	≤ 64	≤ 1	≥4/76
234	1833255	≥ 16	≥ 16	≥ 16	≥ 64	≥ 16	≤ 64	≤ 1	≥4/76
235	1833931	≥ 16	≥ 16	≥ 16	≥ 64	≥ 16	≥ 256	≤ 1	≥4/76
236	1834695	≥ 16	≥ 16	≥ 16	≥ 64	≥ 16	≥ 256	≤ 1	≥4/76
237	1836111	≥ 16	≥ 16	≥ 16	≥ 64	≥ 16	≤ 64	≤ 1	≥4/76
238	1836927	≥ 16	≥ 16	≥ 16	≥ 64	≥ 16	≥ 256	≤ 1	≥4/76
239	1837981	≥ 16	≥ 16	≥ 16	≥ 64	≥ 16	≥ 256	≤ 1	≥4/76
240	1839209	≥ 16	≥ 16	≥ 16	≥ 64	≥ 16	≥ 256	≤ 1	≥4/76
241	1843688	≥ 16	≥ 16	≥ 16	≥ 64	≥ 16	≤ 64	≤ 1	≥4/76
242	1844885	≥ 16	≥ 16	≥ 16	≥ 64	≥ 16	≥ 256	≤ 1	≥4/76
243	1848366	≥ 16	≥ 16	≥ 16	≥ 64	≥ 16	≥ 256	≤ 1	≥4/76
244	1848507	≥ 16	≥ 16	≥ 16	≥ 64	≥ 16	≤ 64	≤ 1	≥4/76

Table4				
Number	Patients ID	Ertapenem	Imipenem	Meropenem
1	1211008	⩾ 2	⩾ 4	⩾ 4
2	1255707	⩾ 2	⩾ 4	⩾ 4
3	1387145	⩾ 2	⩾ 4	⩾ 4
4	1435940	⩾ 2	⩾ 4	⩾ 4
5	1457348	⩾ 2	⩾ 4	⩾ 4
6	1467859	⩾ 2	⩾ 4	⩽ 2
7	1603858	⩾ 2	⩾ 4	⩾ 4
8	1628916	⩾ 2	⩾ 4	⩾ 4
9	1631286	⩾ 2	⩾ 4	⩾ 4
10	1643835	⩾ 2	⩾ 4	⩾ 4
11	1654306	⩾ 2	⩾ 4	⩾ 4
12	1655141	⩾ 2	⩾ 4	⩾ 4
13	1673544	⩾ 2	⩾ 4	⩾ 4
14	1674016	⩾ 2	⩾ 4	⩾ 4
15	1676693	⩾ 2	⩾ 4	⩾ 4
16	1676967	⩾ 2	⩾ 4	⩾ 4
17	1677363	⩾ 2	⩾ 4	⩾ 4
18	1677521	⩾ 2	⩾ 4	⩾ 4
19	1678002	⩾ 2	⩾ 4	⩾ 4
20	1678087	⩾ 2	⩾ 4	⩾ 4
21	1678329	⩾ 2	⩾ 4	⩾ 4
22	1679510	⩾ 2	⩾ 4	⩾ 4
23	1681169	⩾ 2	⩾ 4	⩾ 4
24	1681287	⩾ 2	⩾ 4	⩾ 4
25	1681407	⩾ 2	⩾ 4	⩾ 4
26	1681610	⩾ 2	⩾ 4	⩾ 4
27	1683217	⩾ 2	⩾ 4	⩾ 4
28	1683428	⩾ 2	⩾ 4	⩾ 4
29	1683853	⩾ 2	⩾ 4	⩾ 4
30	1683893	⩾ 2	⩾ 4	⩾ 4
31	1684044	⩾ 2	⩾ 4	⩾ 4
32	1684190	⩾ 2	⩾ 4	⩾ 4
33	1684510	⩾ 2	⩾ 4	⩾ 4
34	1684821	⩾ 2	⩾ 4	⩾ 4
35	1685440	⩾ 2	⩾ 4	⩾ 4
36	1687366	⩾ 2	⩾ 4	⩾ 4
37	1688971	⩾ 2	⩾ 4	⩾ 4
38	1689120	⩾ 2	⩾ 4	⩾ 4
39	1689894	⩾ 2	⩾ 4	⩾ 4
40	1690370	⩾ 2	⩾ 4	⩾ 4
41	1691389	⩾ 2	⩾ 4	⩾ 4
42	1692011	⩾ 2	⩾ 4	⩾ 4
43	1695674	⩾ 2	⩾ 4	⩾ 4
44	1696129	⩾ 2	⩾ 4	⩾ 4
45	1696670	⩾ 2	⩾ 4	⩽ 2
46	1698113	⩾ 2	⩾ 4	⩾ 4
47	1700328	⩾ 2	⩾ 4	⩾ 4
48	1700934	⩾ 2	⩾ 4	⩾ 4
49	1702475	⩾ 2	⩾ 4	⩾ 4
50	1703413	⩾ 2	⩾ 4	⩾ 4
51	1704192	⩾ 2	⩾ 4	⩾ 4
52	1706017	⩾ 2	⩾ 4	⩾ 4

53	1707501	⩾ 2	⩾ 4	⩾ 4
54	1707627	⩾ 2	⩾ 4	⩾ 4
55	1710844	⩾ 2	⩾ 4	⩾ 4
56	1711345	⩾ 2	⩾ 4	⩾ 4
57	1711559	⩾ 2	⩾ 4	⩾ 4
58	1711905	⩾ 2	⩾ 4	⩾ 4
59	1712856	⩾ 2	⩾ 4	⩾ 4
60	1713261	⩾ 2	⩾ 4	⩾ 4
61	1713952	⩾ 2	⩾ 4	⩾ 4
62	1714960	⩾ 2	⩾ 4	⩾ 4
63	1715473	⩾ 2	⩾ 4	⩾ 4
64	1716196	⩾ 2	⩾ 4	⩾ 4
65	1717117	⩾ 2	⩾ 4	⩾ 4
66	1717330	⩾ 2	⩾ 4	⩾ 4
67	1718209	⩾ 2	⩾ 4	⩾ 4
68	1718292	⩾ 2	⩾ 4	⩾ 4
69	1718861	⩾ 2	⩾ 4	⩾ 4
70	1719002	⩾ 2	⩾ 4	⩾ 4
71	1719585	⩾ 2	⩾ 4	⩾ 4
72	1720545	⩾ 2	⩾ 4	⩾ 4
73	1722263	⩾ 2	⩾ 4	⩾ 4
74	1722520	⩾ 2	⩾ 4	⩾ 4
75	1722588	⩾ 2	⩾ 4	⩾ 4
76	1722653	⩾ 2	⩾ 4	⩾ 4
77	1722820	⩾ 2	⩾ 4	⩾ 4
78	1723040	⩾ 2	⩾ 4	⩾ 4
79	1724993	⩾ 2	⩾ 4	⩾ 4
80	1725517	⩾ 2	⩾ 4	⩾ 4
81	1725783	⩾ 2	⩾ 4	⩾ 4
82	1727646	⩾ 2	⩾ 4	⩾ 4
83	1728409	⩾ 2	⩾ 4	⩾ 4
84	1728979	⩾ 2	⩾ 4	⩾ 4
85	1729216	⩾ 2	⩾ 4	⩾ 4
86	1729494	⩾ 2	⩾ 4	⩾ 4
87	1732456	⩾ 2	⩾ 4	⩾ 4
88	1732972	⩾ 2	⩾ 4	⩾ 4
89	1733175	⩾ 2	⩾ 4	⩾ 4
90	1733558	⩾ 2	⩾ 4	⩾ 4
91	1735030	⩾ 2	⩾ 4	⩾ 4
92	1736316	⩾ 2	⩾ 4	⩾ 4
93	1736478	⩾ 2	⩾ 4	⩾ 4
94	1736545	⩾ 2	⩾ 4	⩾ 4
95	1736849	⩾ 2	⩾ 4	⩾ 4
96	1737908	⩾ 2	⩾ 4	⩾ 4
97	1738572	⩾ 2	⩾ 4	⩾ 4
98	1738637	⩾ 2	⩾ 4	⩽ 2
99	1739659	⩾ 2	⩾ 4	⩾ 4
100	1739709	⩾ 2	⩾ 4	⩾ 4
101	1740093	⩾ 2	⩾ 4	⩾ 4
102	1740266	⩾ 2	⩾ 4	⩾ 4
103	1741070	⩾ 2	⩾ 4	⩾ 4
104	1746779	⩾ 2	⩾ 4	⩾ 4
105	1748811	⩾ 2	⩾ 4	⩾ 4
106	1749608	⩾ 2	⩾ 4	⩾ 4

107	1749979	≥ 2	≥ 4	≥ 4
108	1750181	≥ 2	≥ 4	≥ 4
109	1750600	≥ 2	≥ 4	≥ 4
110	1750762	≥ 2	≥ 4	≥ 4
111	1751643	≥ 2	≥ 4	≥ 4
112	1752526	≥ 2	≥ 4	≥ 4
113	1753504	≥ 2	≥ 4	≥ 4
114	1753816	≥ 2	≥ 4	≥ 4
115	1755284	≥ 2	≥ 4	≥ 4
116	1755340	≥ 2	≥ 4	≥ 4
117	1756051	≥ 2	≥ 4	≥ 4
118	1756142	≥ 2	≥ 4	≥ 4
119	1757201	≥ 2	≥ 4	≥ 4
120	1757736	≥ 2	≥ 4	≥ 4
121	1758330	≥ 2	≥ 4	≥ 4
122	1758395	≥ 2	≥ 4	≥ 4
123	1758448	≥ 2	≥ 4	≥ 4
124	1759555	≥ 2	≥ 4	≥ 4
125	1759817	≥ 2	≥ 4	≥ 4
126	1760643	≥ 2	≥ 4	≥ 4
127	1761292	≥ 2	≥ 4	≥ 4
128	1761352	≥ 2	≥ 4	≥ 4
129	1762617	≥ 2	≥ 4	≥ 4
130	1762843	≥ 2	≥ 4	≥ 4
131	1763053	≥ 2	≥ 4	≥ 4
132	1763080	≥ 2	≥ 4	≥ 4
133	1765040	≥ 2	≥ 4	≥ 4
134	1765508	≥ 2	≥ 4	≥ 4
135	1765923	≥ 2	≥ 4	≥ 4
136	1766094	≥ 2	≥ 4	≥ 4
137	1766123	≥ 2	≥ 4	≥ 4
138	1766176	≥ 2	≥ 4	≥ 4
139	1766198	≥ 2	≥ 4	≥ 4
140	1766489	≥ 2	≥ 4	≥ 4
141	1767244	≥ 2	≥ 4	≥ 4
142	1767348	≥ 2	≥ 4	≥ 4
143	1768100	≥ 2	≥ 4	≥ 4
144	1769562	≥ 2	≥ 4	≥ 4
145	1769847	≥ 2	≥ 4	≥ 4
146	1770111	≥ 2	≥ 4	≥ 4
147	1770177	≥ 2	≥ 4	≥ 4
148	1770730	≥ 2	≥ 4	≥ 4
149	1771549	≥ 2	≥ 4	≥ 4
150	1772176	≥ 2	≥ 4	≥ 4
151	1772963	≥ 2	≥ 4	≥ 4
152	1773213	≥ 2	≥ 4	≥ 4
153	1773735	≥ 2	≥ 4	≥ 4
154	1774722	≥ 2	≥ 4	≥ 4
155	1775047	≥ 2	≥ 4	≥ 4
156	1775557	≥ 2	≥ 4	≥ 4
157	1775768	≥ 2	≥ 4	≥ 4
158	1776183	≥ 2	≥ 4	≥ 4
159	1776245	≥ 2	≥ 4	≥ 4
160	1776929	≥ 2	≥ 4	≥ 4

161	1777669	≥ 2	≥ 4	≥ 4
162	1778511	≥ 2	≥ 4	≥ 4
163	1779476	≥ 2	≥ 4	≥ 4
164	1779744	≥ 2	≥ 4	≥ 4
165	1780136	≥ 2	≥ 4	≥ 4
166	1780855	≥ 2	≥ 4	≥ 4
167	1781129	≥ 2	≥ 4	≥ 4
168	1782896	≥ 2	≥ 4	≥ 4
169	1783150	≥ 2	≥ 4	≥ 4
170	1785644	≥ 2	≥ 4	≥ 4
171	1786611	≥ 2	≥ 4	≥ 4
172	1786730	≥ 2	≥ 4	≥ 4
173	1787863	≥ 2	≥ 4	≥ 4
174	1788964	≥ 2	≥ 4	≥ 4
175	1789110	≥ 2	≥ 4	≥ 4
176	1789592	≥ 2	≥ 4	≥ 4
177	1789827	≥ 2	≥ 4	≥ 4
178	1789833	≥ 2	≥ 4	≥ 4
179	1790155	≥ 2	≥ 4	≥ 4
180	1793330	≥ 2	≥ 4	≥ 4
181	1795261	≥ 2	≥ 4	≥ 4
182	1796385	≥ 2	≥ 4	≥ 4
183	1796449	≥ 2	≥ 4	≥ 4
184	1798073	≥ 2	≥ 4	≥ 4
185	1798991	≥ 2	≥ 4	≥ 4
186	1800441	≥ 2	≥ 4	≥ 4
187	1800637	≥ 2	≥ 4	≥ 4
188	1802164	≥ 2	≥ 4	≥ 4
189	1802353	≥ 2	≥ 4	≥ 4
190	1803598	≥ 2	≥ 4	≥ 4
191	1807531	≥ 2	≥ 4	≥ 4
192	1809155	≥ 2	≥ 4	≥ 4
193	1809300	≥ 2	≥ 4	≥ 4
194	1809481	≥ 2	≥ 4	≥ 4
195	1811703	≥ 2	≥ 4	≥ 4
196	1812416	≥ 2	≥ 4	≥ 4
197	1812894	≥ 2	≥ 4	≥ 4
198	1813076	≥ 2	≥ 4	≥ 4
199	1815884	≥ 2	≥ 4	≥ 4
200	1816127	≥ 2	≥ 4	≥ 4
201	1817365	≥ 2	≥ 4	≥ 4
202	1819146	≥ 2	≥ 4	≥ 4
203	1819758	≥ 2	≥ 4	≥ 4
204	1820477	≥ 2	≥ 4	≥ 4
205	1821991	≥ 2	≥ 4	≥ 4
206	1822057	≥ 2	≥ 4	≤ 2
207	1822430	≥ 2	≥ 4	≥ 4
208	1822599	≥ 2	≥ 4	≥ 4
209	1823146	≥ 2	≥ 4	≥ 4
210	1824014	≥ 2	≥ 4	≥ 4
211	1824858	≥ 2	≥ 4	≥ 4
212	1825262	≥ 2	≥ 4	≥ 4
213	1825889	≥ 2	≥ 4	≥ 4
214	1826017	≥ 2	≥ 4	≥ 4

215	1826067	≥ 2	≥ 4	≥ 4
216	1826174	≥ 2	≥ 4	≥ 4
217	1826332	≥ 2	≥ 4	≥ 4
218	1826389	≥ 2	≥ 4	≥ 4
219	1826466	≥ 2	≥ 4	≥ 4
220	1826503	≥ 2	≥ 4	≥ 4
221	1826507	≥ 2	≥ 4	≥ 4
222	1826858	≥ 2	≥ 4	≥ 4
223	1827255	≥ 2	≥ 4	≥ 4
224	1827473	≥ 2	≥ 4	≥ 4
225	1827500	≥ 2	≥ 4	≥ 4
226	1828232	≥ 2	≥ 4	≥ 4
227	1829427	≥ 2	≥ 4	≥ 4
228	1829496	≥ 2	≥ 4	≥ 4
229	1829525	≥ 2	≥ 4	≥ 4
230	1830110	≥ 2	≥ 4	≥ 4
231	1831141	≥ 2	≥ 4	≥ 4
232	1832787	≥ 2	≥ 4	≥ 4
233	1833087	≥ 2	≥ 4	≥ 4
234	1833255	≥ 2	≥ 4	≥ 4
235	1833931	≥ 2	≥ 4	≥ 4
236	1834695	≥ 2	≥ 4	≥ 4
237	1836111	≥ 2	≥ 4	≥ 4
238	1836927	≥ 2	≥ 4	≥ 4
239	1837981	≥ 2	≥ 4	≥ 4
240	1839209	≥ 2	≥ 4	≥ 4
241	1843688	≥ 2	≥ 4	≥ 4
242	1844885	≥ 2	≥ 4	≥ 4
243	1848366	≥ 2	≥ 4	≥ 4
244	1848507	≥ 2	≥ 4	≥ 4
